# Decline in wild bee species richness associated with honey bee (*Apis mellifera* L.) abundance in an urban ecosystem

**DOI:** 10.7717/peerj.14699

**Published:** 2023-02-03

**Authors:** Gail MacInnis, Etienne Normandin, Carly D. Ziter

**Affiliations:** 1 Biology Department, Concordia University, Montreal, Quebec, Canada; 2 Institut de Recherche en Biologie Végétale, University of Montreal, Montreal, Quebec, Canada

**Keywords:** Wild bees, Pollinators, Exploitative competition, Beekeeping, Urban, Biodiversity, Native bees, Honey bees

## Abstract

The spatial heterogeneity of urban landscapes, relatively low agrochemical use, and species-rich floral communities often support a surprising diversity of wild pollinators in cities. However, the management of Western honey bees (*Apis mellifera* L.) in urban areas may represent a new threat to wild bee communities. Urban beekeeping is commonly perceived as an environmentally friendly practice or a way to combat pollinator declines, when high-density beekeeping operations may actually have a negative influence on native and wild bee populations through floral resource competition and pathogen transmission. On the Island of Montréal, Canada there has been a particularly large increase in beekeeping across the city. Over the years following a large bee diversity survey ending in 2013, there was an influx of almost three thousand honey bee colonies to the city. In this study, we examined the wild bee communities and floral resources across a gradient of honey bee abundances in urban greenspaces in 2020, and compared the bee communities at the same sites before and after the large influx of honey bees. Overall, we found a negative relationship between urban beekeeping, pollen availability, and wild bee species richness. We also found that honey bee abundance had the strongest negative effect on small (inter-tegular span <2.25 mm) wild bee species richness. Small bee species may be at higher risk in areas with abundant honey bee populations as their limited foraging range may reduce their access to floral resources in times of increased competition. Further research on the influence of urban beekeeping on native and wild pollinators, coupled with evidence-based beekeeping regulations, is essential to ensure cities contain sufficient resources to support wild bee diversity alongside managed honey bees.

## Introduction

Amidst growing concerns around pollinator declines, cities are increasingly recognized as a potential refuge for wild bee species (Hall et al., 2017). Although wild bee species declines are largely driven by human land use changes, including urbanization (LeBuhn & Luna, 2021), cities can harbor a surprising diversity of pollinators when compared to nearby agricultural or rural systems (Kaluza et al., 2016; Hall et al., 2017; Banaszak-Cibicka et al., 2018). The heterogeneity of the urban landscape, the diversity of greenspace types, and the pesticide-free bylaws in many cities can enhance species richness in urban bee communities. Urban gardens, parks and residential yards provide an especially valuable mix of foraging and nesting resources to support wild bees in cities (Ayers & Rehan, 2021; Matteson, Ascher & Langellotto, 2008; Baldock et al., 2019; Dylewski, Ma’ckowiak & Banaszak-Cibicka, 2020). Urban bee communities often include solitary and social species, cavity and ground nesters, many pollen generalists and occasionally pollen specialists (Hernandez, Frankie & Thorp, 2009; Normandin et al., 2017). This diversity of urban bees is essential to maintain ecosystem health and resilience through their role in pollination, and as hosts and prey for other species (Kleijn et al., 2015). Increasing public attention on the existence and importance of wild urban bees provides a great opportunity for biodiversity conservation in cities. However, the Western honey bee (*Apis mellifera* L.) is oddly perceived as a symbol of biodiversity conservation by many citizens, the mass media, and institutions across the globe (Smith & Saunders, 2016; Van Vierssen Trip et al., 2020).

There has been a rapid increase in the number of honey bee colonies introduced to many cities, often unregulated and endorsed by public authorities in a misdirected effort to mitigate pollinator declines (Lorenz & Stark, 2015). This increase in hobby and commercial beekeepers has produced high densities of colonies in many cities, sometimes on the order of six or more colonies per km^2^ (Ropars et al., 2019; Casanelles-Abella & Moretti, 2022). As healthy colonies can contain upwards of 50,000 bees by midsummer (Sagili & Burgett, 2011), this has the potential to substantially increase the competitive pressure on native bees for a limited amount of pollen and nectar provided by the urban flora. High-density beekeeping operations may also have a negative influence on wild bees and other honey bees through increased parasite and pathogen transmission (Geldmann & González-Varo, 2018; Geslin et al., 2017).

Though more studies are beginning to show that over-abundant honey bee populations negatively impact urban wild bee diversity and disrupt plant–pollinator networks (Lorenz & Stark, 2015; Plascencia & Philpott, 2017; Renner et al., 2021; Ropars et al., 2019; Su et al., 2022), there is no consensus on the impact of mass introductions of honeybee colonies on the urban wild bee community (reviewed by Mallinger, Gaines-Day & Gratton, 2017 and Wojcik et al., 2018). The effects of resource (exploitative) competition by introduced species on wild bee taxa is difficult to predict, as it is difficult to disentangle the influence of habitat and floral resource loss from competition and foraging habits and ecological traits vary widely across bee families, genera, and species (see Prendergast, Dixon & Bateman, 2021). Competition may be highest when there is the greatest niche overlap among taxa, with generalist bees more likely to directly compete for resources with honey bees. However, pollen specialists may suffer the most detrimental effects as specialist species cannot alternate host plants (Wasser & Ollerton, 2006). As urban bees are largely generalist species, ecological traits at finer scales may be more relevant, such as body size and energy requirements. Small-bodied bees generally have smaller foraging ranges than large bees (Greenleaf et al., 2007); bees with smaller foraging ranges have reduced ability to travel great distances in search of food and may suffer more negative consequences in times of increased competition (Herbertsson et al., 2016). However, large bees require more floral resources as their energetic costs of foraging are higher, and they may leave areas of increased competition in search of adequate sustenance (Goulson, Stout & Kells, 2002). Further research on the competitive interactions between managed and wild bees and the influence of functional traits is essential to increase our understanding of the effects of interspecies competition on biodiversity in cities.

Exploitative competition for floral resources is thought to be more probable than direct interference competition among bees (Cane & Tepedino, 2016). Thus, the composition of the floral community has a substantial effect on the level of competition realized by bee communities. However, there is great variability in the richness and abundance of floral resources across urban landscapes. Though urban plant communities are diverse, they are often small and fragmented, and consist of highly cultivated plants, bred for large floral displays or other aesthetic properties rather than for pollen or nectar content (Lowenstein & Minor, 2016). The influence of the floral community on the structure of urban bee communities may differ from that of natural or rural environments, and plant–pollinator relationships in non-urban studies likely cannot be generalized to cities (Dylewski, Ma’ckowiak & Banaszak-Cibicka, 2020; Prendergast & Ollerton, 2021). Spatiotemporal analyses of floral resources and their value to bees are needed to assess the influence of the urban floral community on the bee community, and to ensure urban environments contain sufficient resources to sustain managed honey bees and a diversity of wild bee species.

In this study, we examined the wild bee communities at urban green spaces spanning a gradient of honey bee abundances, and before and after the urban beekeeping boom in Montréal, Canada. Bee diversity surveys conducted on the Island of Montréal in 2012 and 2013 (7 and 8 years prior to our study) provided a unique opportunity to study the effects of honey bee abundance on the urban wild bee community (Normandin et al., 2017). Over the years following these surveys, there has been a large influx of honey bee colonies. The number of colonies has gone from 238 in 2013 to almost 3,000 colonies in 2020 (Ministère de l’Agriculture, des Pêcheries et de l’Alimentation - MAPAQ, G. Claing, 2019, pers. comm.) largely due to the popularization of urban beekeeping. The original bee surveys provide a baseline to compare wild bee communities across time, as they were conducted when honey bee presence was minimal, and sampling locations now exist along a gradient of honey bee abundance. Using a natural experimental approach, the central aims of this study were to: (1) examine the species richness and composition of wild bee communities along a gradient of honey bee abundances; (2) assess the influence of honey bee abundance on floral resource (pollen) availability; and (3) compare the richness and composition of the wild bee communities before and after the large increase in the honey bee colonies in Montréal. We hypothesized that wild bee species richness would decrease with honey bee abundance across sites and years due to exploitative competition, and that bee body size would influence species responses to competition by honey bees. As the limited forage range of smaller bees may reduce access to floral resources in times of increased competition, we tested the influence of honey bee abundance on small *versus* large bee richness and abundance. We also examined whether increased honey bee presence had a differential effect on native *versus* exotic bee richness and abundance, as native bees are generally of most conservation concern.

## Methods

## Study sites and sampling protocol

This research was conducted at 15 sites across the Island of Montréal, Quebec, Canada in 2020. The access to field sites was authorized by the City of Montréal—Direction de la Gestion des Parcs et de la Biodiversité, and verbally by the managers of community gardens and cemeteries. To facilitate comparison with baseline measurements (Normandin et al., 2017), sites were selected to match those sampled in 2013. We originally planned to sample at 21 sites but the Covid-19 pandemic restricted access to six sites. We were also unable to access any sites in the early season (May) due to pandemic-related restrictions on field permits. Additionally, two high honey bee abundance sites were added in 2020 that were not sampled in 2013. The selected sites spanned a gradient of honey bee densities, as estimated based on data provided by the Ministère de l’Agriculture, des Pêcheries et de l’Alimentation du Québec (MAPAQ) and through personal communications with local beekeeping organizations. Sites spanned urban green space types known to attract pollinators, including nine community gardens which grew a variety of fruit and vegetables, four large cemeteries, and two large nature parks. The cemeteries and parks were lower in floral richness, but contained larger expanses of uncultivated flowering plant species than garden sites; *e.g.*, goldenrod (*Solidago canadensis*) and clover (*Trifolium repens*). All sites were at least 1.5 km apart, exceeding the foraging range of most wild bees in our area, with the exception of *Bombus* which has been known to forage at distances above 1 km (Gathmann & Tscharntke, 2002; Goulson & Stout, 2001; Hagen, Wikelski & Kissling, 2011).

**Figure 1:**
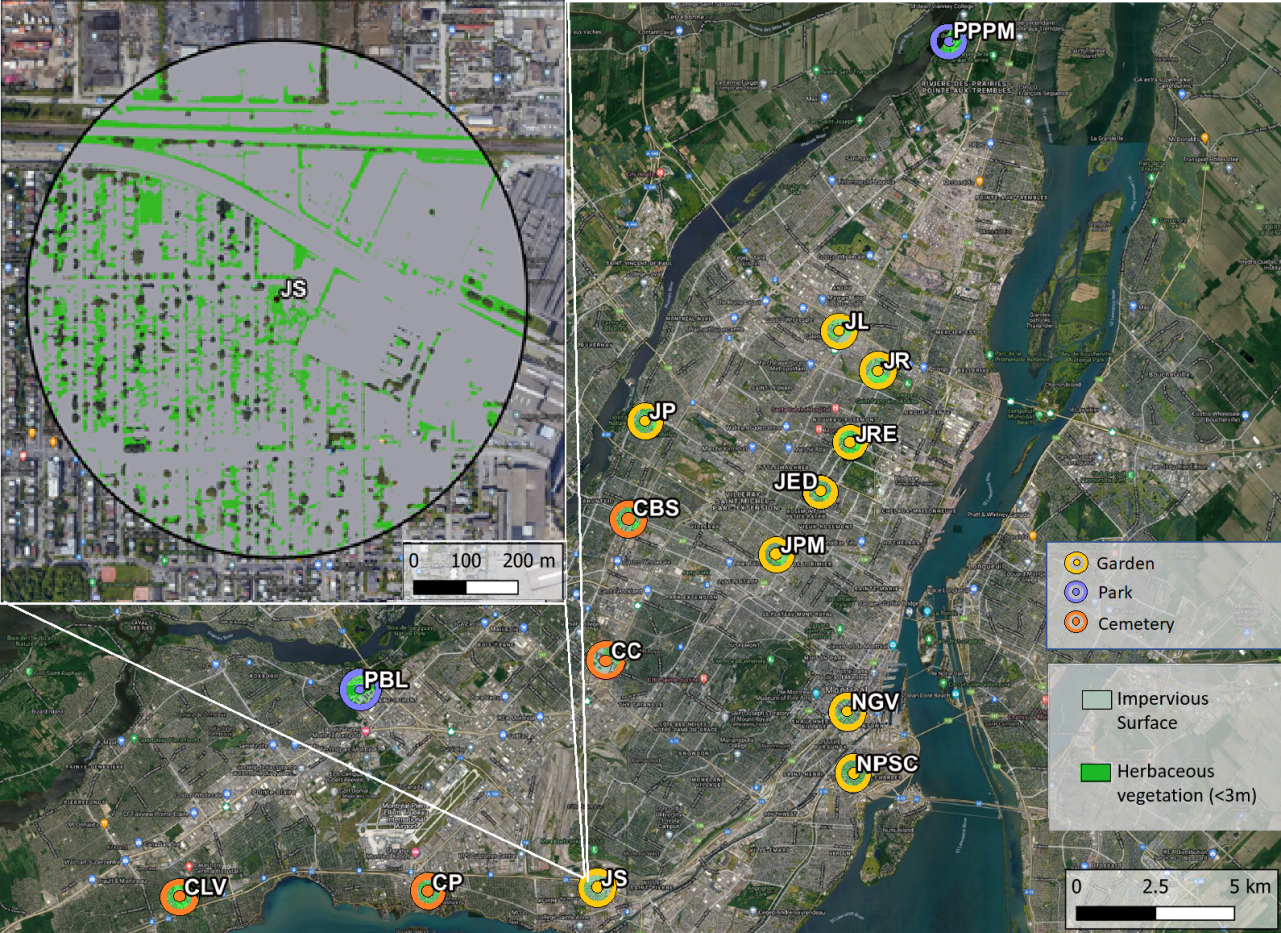
Map of study sites across the Island of Montréal, Quebec, Canada. Herbaceous vegetation was quantified as greenery under three meters in height, impervious surface was the area of pavement and built infrastructure. Top left is an enlarged view highlighting the herbaceous vegetation (bright green) and impervious surface (grey) in 500 meter buffers around each site. Abbreviated site names are adjacent to each site. Map Base layer: Google ©2019, Map data: ©Montreal Metropolitan Community, 2011–2021.

For each site, we calculated the proportion of impervious surface and herbaceous greenery within a 500 m radius as proxies for the variation in habitat and floral resource availability in the broader landscape. Herbaceous greenery was quantified as the area of green vegetation of less than 3 m height, as it has been positively correlated to wild bee diversity (Fortel et al., 2014; Matteson, Grace & Minor, 2013; Lowenstein et al., 2014). Impervious surface is generally uninhabitable for wild bees and has been shown to negatively affect bee diversity in urban areas (Wenzel et al., 2020). A 500 m buffer was chosen as it encompasses the foraging rage of most wild bee species in our area (Gathmann & Tscharntke, 2002; Greenleaf et al., 2007). Using satellite imagery at 1 m resolution (L’Indice Canopée Métropolitain, Communauté Métropolitaine de Montréal) and QGIS v3.10 (QGIS Development Team, 2021) we calculated average impervious surface cover and area of herbaceous greenery at each of our sites. Impervious surface included built infrastructure and paved surfaces. The area of impervious surface was on average 58% across sites, but spanned a range of values from 24% to 85%. The average herbaceous greenery was 25%, with a minimum of 7% and a maximum of 43% across sites (see map in Fig. 1).

### Bee community sampling

All bee sampling was done on warm (>18 °C), sunny days with little wind (<2 m/s) from 0900 h to 1630 h in the spring and summer (between June 18 and Sept 10, 2020). Sites were divided into “Eastern” and “Western” routes to facilitate ease of sampling. Sampling plots were located within a 100 × 100 m area in the centre of each site, at the location central to the bee sampling plots in 2012 and 2013 of Normandin et al. (2017). Eight pan trap triplets (24 pans total per site) were distributed throughout this area, elevated to the average height of most of the flowering plants (0.5 m). All pan trap triplets were placed at least 10 m apart to reduce inter-trap competition (Droege et al., 2010).

The bee sampling protocol for both routes involved four consecutive days of sampling, such that each route was sampled over two days. Pan traps were installed on the Eastern route on the first day of sampling and pan traps were installed on the Western route on the following day. On the third day of sampling, pan traps from Eastern route were collected by one observer (48 hrs of passive sampling) while timed aerial netting was conducted by the same two observers at each site. Aerial netting consisted of 5 min of sweep netting by each of the two observers, targeting bees on open flowers at each of the eight plots at each site (10 min of sweeping total per site). Timing was stopped for each collection event and resumed when the observer was ready to continue searching for bees. Ten minutes of active sampling was chosen to remain consistent with the length of time allocated to netting in the bee diversity surveys done at the same sites in 2013. All netted and pan trapped bees were returned to the lab for identification under the microscope. Honey bee abundance at each site was quantified by counting all honey bees seen foraging on flowers with a tally counter during the 10 min aerial netting periods, as accurate data on managed honey bee hive densities and locations were not available at local scales. On the final day of sampling, the pan traps on the Western route were collected, and the netting and honey bee counting protocol was followed exactly as described for the Eastern route.

This sampling regime was repeated approximately every three weeks for a total of five sampling periods per site over the season beginning on June 18, July 8, July 31, August 17 and September 7. The order of site visitation was rotated so that each site received at least two net collections in the morning (0900 h–1130 h), two in the early afternoon (1130 h–1400 h) and one in late afternoon (1400 h–1630 h), as bees are more active in the morning and early afternoon. Where possible, all wild bees were identified to species using Ascher & Pickering (2021), dichotomous keys (Gibbs, 2011; Gibbs et al., 2013; Williams et al., 2014), and the reference specimens at the Ouellet-Robert Entomological Collection at the Biodiversity Centre of the Université de Montréal; voucher specimens are housed at this collection. We use the term “wild bees” to refer to all non-managed bees, which includes both native and exotic species. Where species-level identifications were not possible, individuals were given a morphospecies identifier (eight species total).

To assess whether honey bee abundance had an effect on bees with particular traits, we then classified wild bees as exotic or native species and by bee body size (inter-tegular span—ITS). Exotic or native species status were determined as per Normandin et al. (2017). ITS is a strong predictor of bee body mass and thoracic wing musculature, and thus is correlated with foraging range (Greenleaf et al., 2007). Small bees were classified as those with ITS <2.25 mm and large bees were those with an ITS ≥ 2.25 mm as in Benjamin, Reilly & Winfree (2014). The maximum foraging range of small bees in these size categories would be less than 1 km, whereas large bees would be capable of foraging beyond 1 km from nesting sites. However, typical foraging ranges of both small and large bees would be much less than 1 km, and most solitary bees are heavily dependent on floral resources within a few hundred meters of their nests (Zurbuchen et al., 2010). Very large bees were classified as those with ITS >5 mm. Only bees in the genus *Bombus* fell into this category, their foraging ranges can be much larger than other solitary bee species (*e.g.*, 1–2 km; Walther-Hellwig & Frankl, 2003).

### Floral diversity and pollen surveys

On the first day of sampling on each route and sampling round (five rounds), we measured floral richness and floral density using 2 m × 2 m quadrats centered on the location of each pan trap triplet (eight quadrats) at each site. We identified all flowering plants to the lowest taxonomic level possible (usually species) and visually estimated the proportion of open flowers *versus* ground cover inside the quadrats. To estimate whether honey bee abundance influenced floral resource depletion, we also haphazardly collected 8–10 clover flowers (*Trifolium repens*) at each site for pollen analyses (in sampling plots when possible) during three sampling rounds (June 31, August 17 and September 7). We chose white clover as it was common to all sites and most sampling periods, and its shallow florets are accessible to both honey bees and wild bees (Free, 1993). Though studies show bumble bees are the most common wild pollinator of *T.repens* (Kanduth et al., 2021; Larson, Kesheimer & Potter, 2014; Lundin et al., 2017), in urban environments it can be an important resource for other solitary bees as well (*e.g.*, *Osmia, Lasioglossum* in Hennig & Ghazoul (2011); *Andrena and Halictus* in Wolfin et al., 2021). On each of the clover flowers collected, we counted the number of brown and white florets and chose three brown and three white florets for pollen analysis. While white florets are still receptive to pollinators, wilted (brown) florets could still contain pollen, so we included both brown and white florets in pollen analyses. We separated the florets by color and carefully placed them in centrifuge tubes with 0.5 ml of ethanol for subsequent pollen analysis in the lab. Following Torné-Noguera et al. (2016), we added three drops of a Safranin O stain solution to the centrifuge tubes and sonicated for 10 min each to dislodge pollen. We then pipetted 2.5 µl of solution onto a microscope slide, covered and sealed with clear nail polish. We counted all pollen grains found in the solution at 40x magnification and weighed the remaining volume of solution on an analytical balance. The final pollen counts were a product of the amount of pollen in 2.5 µl, the remaining volume of pollen solution, and the total number of brown and white florets counted on the sample flower.

### Bee communities between years—2020 *vs.* 2013

In addition to assessing the impact of honey bee abundance on wild bee richness in 2020, we compared the 2020 wild bee community to that of 2013 at the same sites sampled by Normandin et al. (2017), when honey bee presence was minimal in Montréal. They also sampled bees in 2012 but bee community comparisons were limited to the more complete 2013 data set. To assess the influence of increased honey bee abundance on wild bee communities, we first needed to estimate the change in honey bee abundance between 2013 to 2020. We used the number caught in pan traps and nets as our 2013 baseline. Fewer than 20 honey bees were collected in each sampling periods in 2013 (mean ± SD = 4.56 ± 7.81). There were 238 hives on the island in 2013 (McCune et al., 2020) and there were 2918 known hives on the island in 2020 (The Ministère de l’Agriculture des Pêcheries et de l’Alimentation—MAPAQ—G Claing, 2019, pers. comm.). Given that honey bee abundance was relatively low in 2013, we then calculated the increase in honey bees between years by subtracting the total number of honey bees detected in 2020 from the number of honey bees caught in net and pan traps in 2013. The 2013 study did not include measures of the floral communities, however, McCune et al. (2020) measured the richness and density of floral communities at the same sites in 2016 and claimed the floral communities did not vary substantially from those in 2013. Thus, we used the 2016 floral community measurements for our comparisons.

### Statistical analyses

#### Wild bee diversity, bee traits and honey bee abundance

We used a suite of diversity metrics to assess the wild bee community: species richness, bee abundance, Pielou’s evenness, Shannon-Wiener and Simpson’s diversity indices. The Shannon index is more sensitive to the presence of rare species whereas Simpson’s index is more weighted towards common species. Pielou’s evenness index provided a comparison of wild bee species dominance with increasing honey bee abundance (Morris et al., 2014). Honey bee abundance was the sum of honey bees detected in nets and pan traps during sampling periods (continuous variable). Samples were pooled across plots and sampling methods for each site, and each metric was calculated for the bee communities at each site and sampling period. Bee abundance refers to the number of bee individuals detected. We calculated raw species richness based on the number of species we detected at each site and sampling period, however sample coverage-based rarefaction was used to compare species richness across sites (iNEXT package, Hsieh, Ma & Chao, 2020; Chao et al., 2014). Coverage-based rarefaction compares species richness among samples of equal sample coverage, based on the lowest percent coverage detected at any site (here, 72%). All diversity indices were also calculated using the iNEXT package. Honey bees were not included in calculations of diversity indices as we were assessing their influence on richness and diversity of the wild bee community.

We used generalized linear mixed models to assess the influence of honey bee abundance on metrics of wild bee diversity (GLMM, package: lme4, Bates et al. (2015) and R Core Team (2021)). We modelled rarefied species richness as Poisson distributed with a log-link function, and Shannon, Simpson and Pielou’s indices with Gaussian distributions with an identity link function. Bee abundances were modelled with a negative binomial distribution due to overdispersion. All models included sampling period, honey bee abundance, floral richness, floral density, greenspace type and the proportion of impervious surface and herbaceous greenery within a 500 m radius as fixed effects, with site as a random effect. For the very large bee models, the proportion of impervious surface and herbaceous vegetation at 1500 m buffers were used to account for the larger foraging range of these bees. All fixed effects were centered and scaled to unit variance to permit comparison of effect sizes. Model fit and overdispersion were assessed through diagnostic tests of model residuals in the DHARMa package (Hartig, 2021) and multicollinearity was assessed using variance inflation factors (“car” package, Fox et al., 2019).

We also used GLMMs to test the influence of honey bee abundance on the richness and abundance of native and exotic bees and of particular size classes. We built separate models for both richness and abundance in each bee trait group. Response variables were the richness and abundance of small (ITS <2.25 mm), large (ITS ≥ 2.25 mm), and very large bees (ITS ≥ 5.0 mm), and native and exotic bees. Small and large bee richness and abundance were negative binomial distributed, while the richness and abundance of very large bees, and native and exotic bees followed a Poisson distribution. Model fit and overdispersion were assessed by using the diagnostic tests of model residuals (“DHARMa” package; Hartig, 2021). Honey bee abundance, floral richness, floral density, sampling period and the proportion of impervious surface and herbaceous vegetation at 500 m buffers around each site were included as fixed effects; site was a random effect.

#### Wild bee community composition

We used NMDS ordination and GLMMs to examine differences in bee community composition among sites that differed in honey bee abundance. We first categorized sites and sampling periods into three groups according to honey bee abundance: low honey bee sites were those with 0 to 19 honey bee individuals detected in sampling periods (*n* = 39); medium honey bee sites were those with 20 to 40 honey bee individuals detected in sampling periods (*n* = 13); high honey bee were those with >40 honey bee individuals detected in sampling periods (*n* = 22). These honey bee abundance categories were only used for ordination analyses, where a categorical variable was necessary. We conducted a sensitivity analysis and these three groupings were ultimately chosen as they resulted in sufficient numbers of sites and sampling periods in each group to allow comparisons. We used a non-metric multidimensional scaling ordination (NMDS, package: vegan, Oksanen et al. (2020)) with Bray-Curtis distances to visualize bee community structure at low, medium and high honey bee sites in 2020. A three-axis solution was used, as it lowered the final stress below 0.20 and additional axes resulted in little improvement.

To further examine the responses of individual bee species to honey bee abundance, we modeled changes in the relative abundance of all species represented by 20 individuals or more with GLMMs using the package ‘mvabund’ with the function ‘manyglm’ (Wang et al., 2012). This function fits a separate generalized linear mixed model to each species in the abundance matrix. We used a Poisson distribution to model the abundance of each species and honey bee abundance, floral richness, floral density, sampling period and the proportion of impervious surface and herbaceous vegetation at 500 m buffers around each site were included as fixed effects in models; site was included as a random effect. The influence of predictor variables on individual species was calculated using likelihood-ratio tests. We also visually examined which species were present and absent at sites that saw the largest increase in honey bee abundance (>40 honey bees detected in 10-minute sampling periods in 2020) since 2013.

#### Wild bee community analyses across years (2013 *vs.* 2020)

We used GLMMs to test the influence of honey bee increase on wild bee richness and bee trait groups across years. We compared rarefied species richness, Shannon, Simpson’s and Pielou’s indices of the 2020 wild bee community to that of 2013 (bees collected by (Normandin et al., 2017). We did not examine the influence of honey bee abundance on wild bee abundance due to differences in sampling effort between years. Honey bee increase was the difference in bees counted in 2020 and those caught in net and pan traps in 2013, as described above. “Year” was added to each model as a fixed effect and the interactive effect of year and the increase in honey bees was tested on each response variable to determine whether honey bee increase between years affected wild bee richness, or richness within bee trait groups. Shannon’s diversity was modeled with a Gaussian distribution and rarified species richness, the richness of native and exotic bees, and the bees of the three body size classes were modeled with a negative-binomial distribution due to overdispersion. All models again included sampling period, floral richness, floral density, greenspace type and the proportion of impervious surface and herbaceous greenery within a 500 meter radius as fixed effects, with site as a random effect. The two high honey bee abundance sites that were added to the study design in 2020, but not sampled in 2013, were not included in the comparative analyses between years.

#### Pollen depletion

To estimate pollen depletion across sites, we modelled the influence of honey bee abundance on pollen content in *T. repens* flowers using GLMMs. Honey bee abundance, wild bee abundance, floral richness, floral density, and sampling period were included as covariates; site was included as random effect. The pollen count data was zero-inflated, so a Tweedie distribution was used. Model fit and overdispersion were assessed by using the diagnostic tests of model residuals (“DHARMa” package; Hartig, 2021).

## Results

We sampled a total of 6,217 bees across 15 sites in 2020 in Montréal, QC. We collected 3,926 wild bees belonging to 22 genera and 120 species, and detected 2,291 honey bees. In comparison, 5,171 wild bees belonging to 163 species were found in 2013, with very few honey bees detected in nets or pan traps (122 individuals). The sample coverage curves showed a similar coverage across sites, but on average, sites with the higher honey bee abundances (mostly community gardens) had slightly higher sample coverage (Fig. S1). We recorded a total of 250 distinct plant species across sites. Overall, the floral richness was higher at community gardens, and these garden sites also harboured the most honey bees (Table S1). See Tables S7 and S8 in supplementary material for full bee and floral species lists.

### Wild bee diversity, bee traits and honey bee abundance

**Figure 2:**
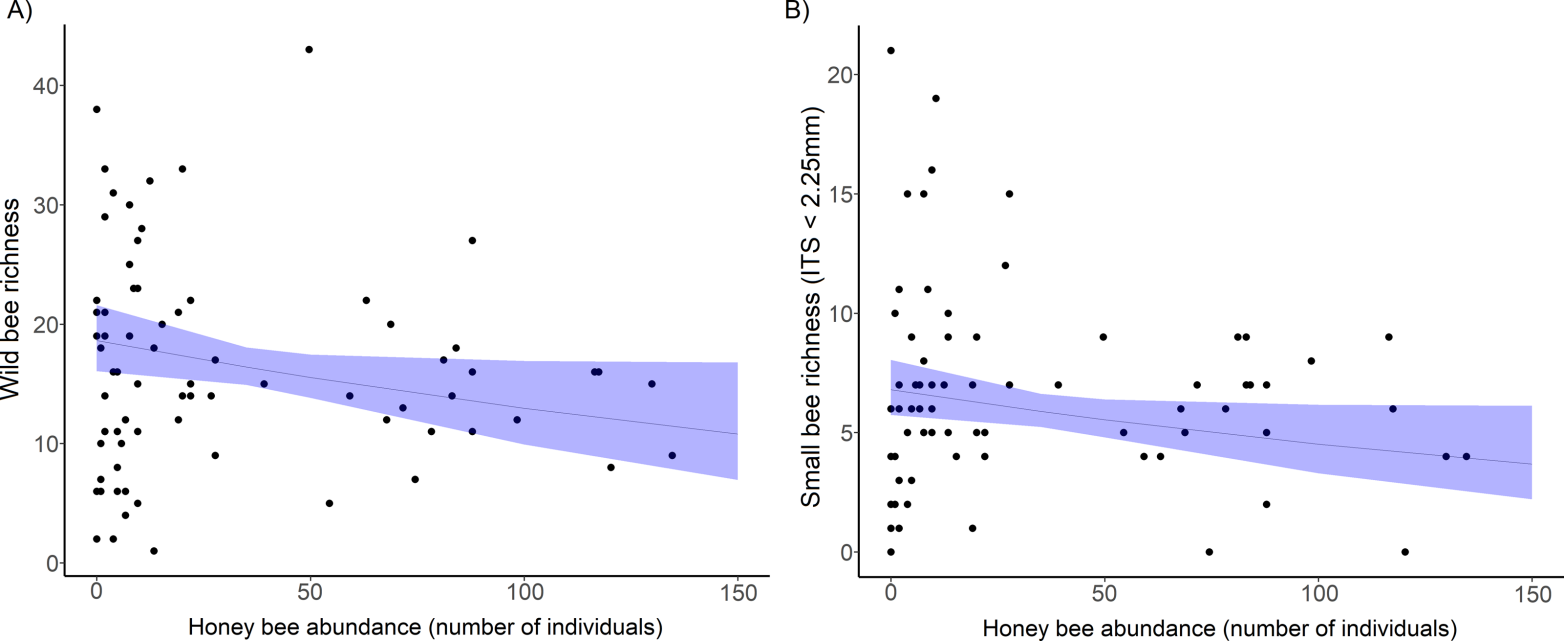
The decline in richness of urban wild bees and small bees with honey bee abundance. (A) Total wild bee richness with increasing honey bee abundance. (B) the effect of honey bee abundance on urban small wild bee richness (inter-tegular span <2.25 mm). Honey bee abundance is the sum of honey bee individuals counted during 10 min netting periods and collected in pan traps at each site and sampling period. Each point is a site and sampling period (5 sampling periods per site). The solid line is the prediction of the GLMM with 95% confidence intervals is shown.

Wild bee species richness declined significantly with an increase in honey bee abundance (Table 1, Fig. 2A), but overall wild bee abundance was not affected by honey bee abundance (${\chi }_{2}^{1}$ = 1.34, *p* = 0.246, Table 1). Shannon’s diversity of the wild bee community decreased significantly with increasing honey bee abundance (*χ*_2_ = 4.02, *p* = 0.044), but honey bee abundance did not influence Simpson’s diversity (*χ*_2_ = 0.19, *p* = 0.655) and Pielou’s evenness (*χ*_2_ = 0.12, *p* = 0.730, see Table S2). Wild bee richness increased with floral richness (*χ*_2_ = , *p* = 0.018) and floral density (*χ*_2_ = , *p* = 0.021; Table 1), but there was no interactive effect of honey bee abundance and flowering plant richness (*χ*^2^ = 2.31, *p* = 0.13) or floral density (*χ*^2^ = 0.88, *p* = 0.35) on wild bee richness.

**Table 1:** Results of GLMM testing of the influence of honey bee abundance on wild bee richness and abundance. Data were pooled across plots at each site and sampling period (*N* = 8 plots per site, 15 sites, 5 sampling periods). Covariates included, floral richness, floral density, greenspace type (park, community garden, cemetery), sampling period, and the proportion of herbaceous vegetation and impervious surface within a 500 m buffer around each site. Floral richness and density were measured locally at each site, and herbaceous vegetation was the amount of green vegetation of >3 m in height within the buffer zone, and was used as a proxy for floral resource availability within the foraging range of wild bees. Predictors for each model were scaled and centered on zero. *β* is the coefficients for each predictor, *CI* is the 95% confidence intervals and *p* is the significance. Significant predictors for either bee richness or abundance are in bold.

	**Wild bee richness**			**Wild bee abundance**			**Small bee richness**		
*Predictors*	*β*	*CI*	*p*	*β*	*CI*	*p*	*β*	*CI*	*p*
(Intercept)	2.46	0.98–3.94	**<0.001**	1.76	−1.53–5.06	0.293	2.71	1.06–4.35	0.001
**Honey bee abundance**	−0.18	−0.36–0.01	**0.043**	−0.07	−0.18–0.05	0.246	−0.20	−0.41—0.00	**0.046**
**Floral richness**	0.40	0.07–0.73	**0.018**	0.48	0.22–0.75	**<0.001**	0.01	−0.37–0.38	0.964
**Floral density**	0.38	0.06–0.70	**0.021**	0.58	0.41–0.75	**<0.001**	0.12	−0.24–0.48	0.506
Herbaceous veg. (%)	0.29	−0.25–0.84	0.291	−0.05	−1.45–1.36	0.947	−0.14	−0.74–0.46	0.649
Impervious surface (%)	−0.24	−1.04–0.55	0.546	0.34	−1.78–2.45	0.756	−0.77	−1.65–0.12	0.089
**Greenspace type (garden)**	−0.29	−0.65–0.07	0.118	0.87	0.21–1.53	**0.010**	0.30	−0.11–0.71	0.158
**Greenspace type (park)**	−0.80	−1.18–0.43	0.968	**<0.001**	0.19–2.08	**0.019**	−0.48	−0.93–0.04	**0.032**
Sampling period (2)	−0.08	−0.54–0.68	0.838	−0.19	−0.47–0.09	0.181	0.35	−0.47–1.17	0.408
**Sampling period (3)**	0.08	−0.68–0.84	0.845	0.34	0.06–0.62	**0.015**	0.02	−0.81–0.84	0.971
Sampling period (4)	0.20	−0.56–0.96	0.604	−0.17	−0.45–0.11	0.238	−0.52	−1.36–0.32	0.221
Sampling period (5)	−0.09	−0.86–0.68	0.824	−0.16	−0.45–0.13	0.273	−0.50	−1.35–0.34	0.244

The bee trait analysis showed that the number of small bee species decreased significantly with honey bee abundance (Fig. 2B, Table 1). Small bee abundance also decreased with honey bee abundance (*χ*_2_ = 3.94, *p* = 0.019). Large bees and bees in the genus *Bombus* (very large bees) were not affected by honey bee abundance (Table S3). We found no influence of honey bee abundance on native bee or exotic bee richness or abundance (Table S3). In the bee trait analysis, the only significant predictor of native bee richness was floral richness (*χ*_2_ = 5.74, *p* = 0.017), and native bee abundance increased significantly with both floral richness (*χ*_2_ = 4.57, *p* = 0.032) and density (*χ*_2_ = 7.64, *p* = 0.005).

### Wild bee community composition

**Figure 3:**
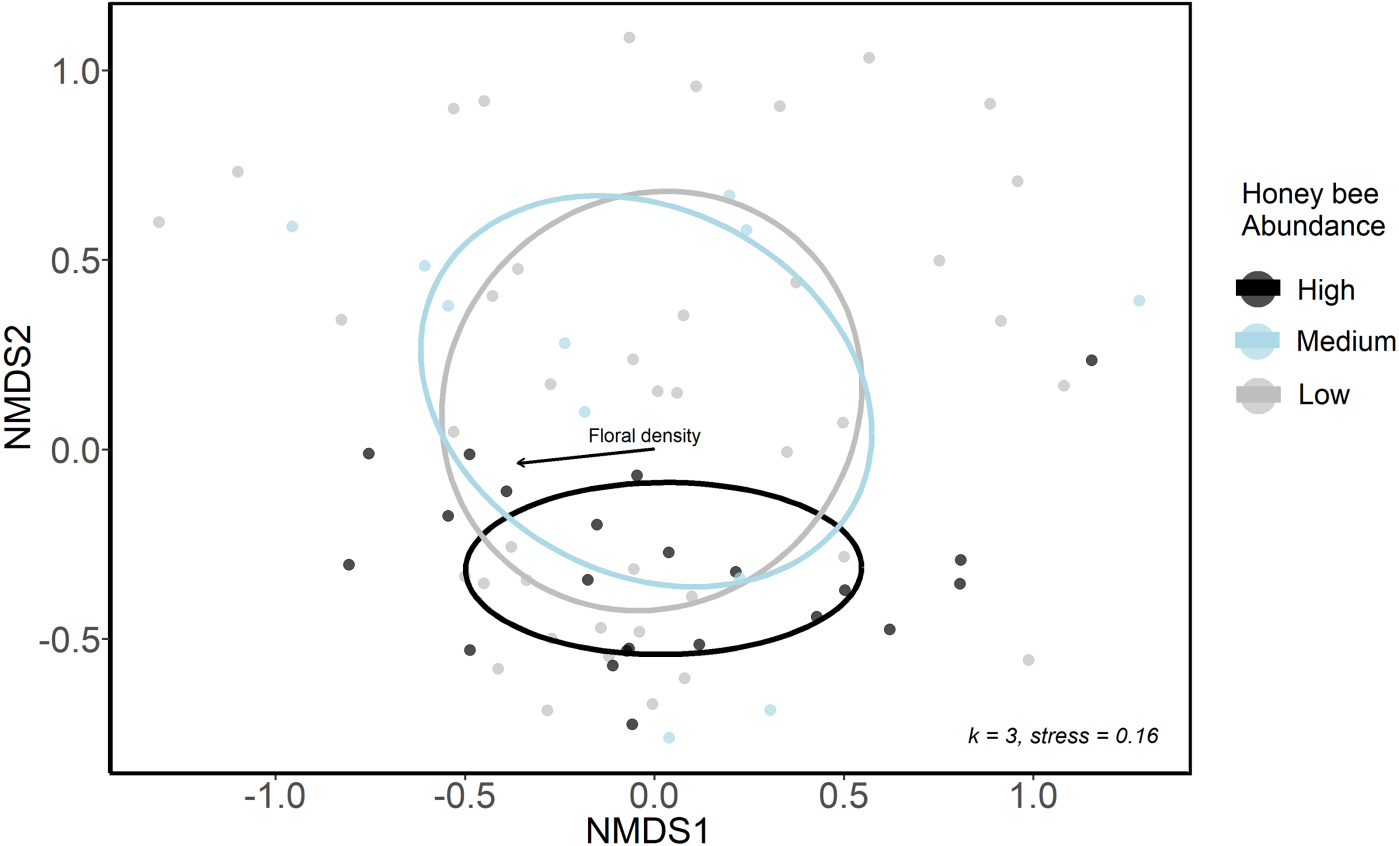
Ordination (NMDS) of urban wild bee communities in urban green spaces. Color indicates honey bee abundance detected during each sampling period. High honey bee abundance sites were classified as those with 40 + honey bees detected during 10-minute net sampling periods, medium honey bee abundance sites had 20–40 honey bees detected, and low honey bee abundance sites had <20 honey bees detected. Environmental variables included the proportion of impervious surface and herbaceous greenery in 500 m buffers of study sites, local floral richness and floral density. The only significant influence on point placement in ordination space was floral density (arrow).

The urban wild bee communities at low and medium honey bee abundance sites were similar and were spread more widely across the first two NMDS axes, whereas sites high in honey bee abundance were closer in ordination space (Fig. 3A). This suggests that the composition of wild bee communities at sites with high honeybee abundance were more similar to each other than the bee communities at sites with lower honey bee abundance. However, low, medium and high honey bee abundance sites did not form distinct communities (ANISOM *R* = 0.001, *p* = 0.47), nor did the bee communities among three greenspace types (ANISOM *R* = 0.03, *p* = 0.26). The differences in the bee communities among sampling periods were statistically significant (ANISOM *R* = 0.20, *p* < 0.001), as expected due to differences in species emergence times and flight seasons. Floral density (Mantel test, *r*^2^ = 0.16, *p* = 0.008) was the only environmental variable with a significant influence on bee community placement along NMDS axes.

When examining individual bee species’ responses, honey bee abundance significantly influenced 8 individual species abundances (*M. rotundata, H. communis, C. mikmaqi, C. calcarata, C. dupla, B.impatiens, B.griseocollis and A. virescens*). However, the effect was positive; most of these species were more abundant at sites with high honey bee abundance (*M. rotundata*, *H. communis, B. impatiens, B. griseocollis and A. virescens*). The influence of honey bee abundance was negative only on the *Ceratina* species, but one site contained over 20 times more *Ceratina* individuals than any other site. When this site was removed from analyses there was no effect of honey bee abundance on *Ceratina* species. When examining individual species presence or absence at high honey bee abundance sites in 2020, there were 13 species that were present at all sites except for those that saw the highest increase in honey bee abundance (>40 individuals) from 2013 to 2020 (Table S4).

### Wild bee community analyses across years (2020 *vs.* 2013)

**Figure 4:**
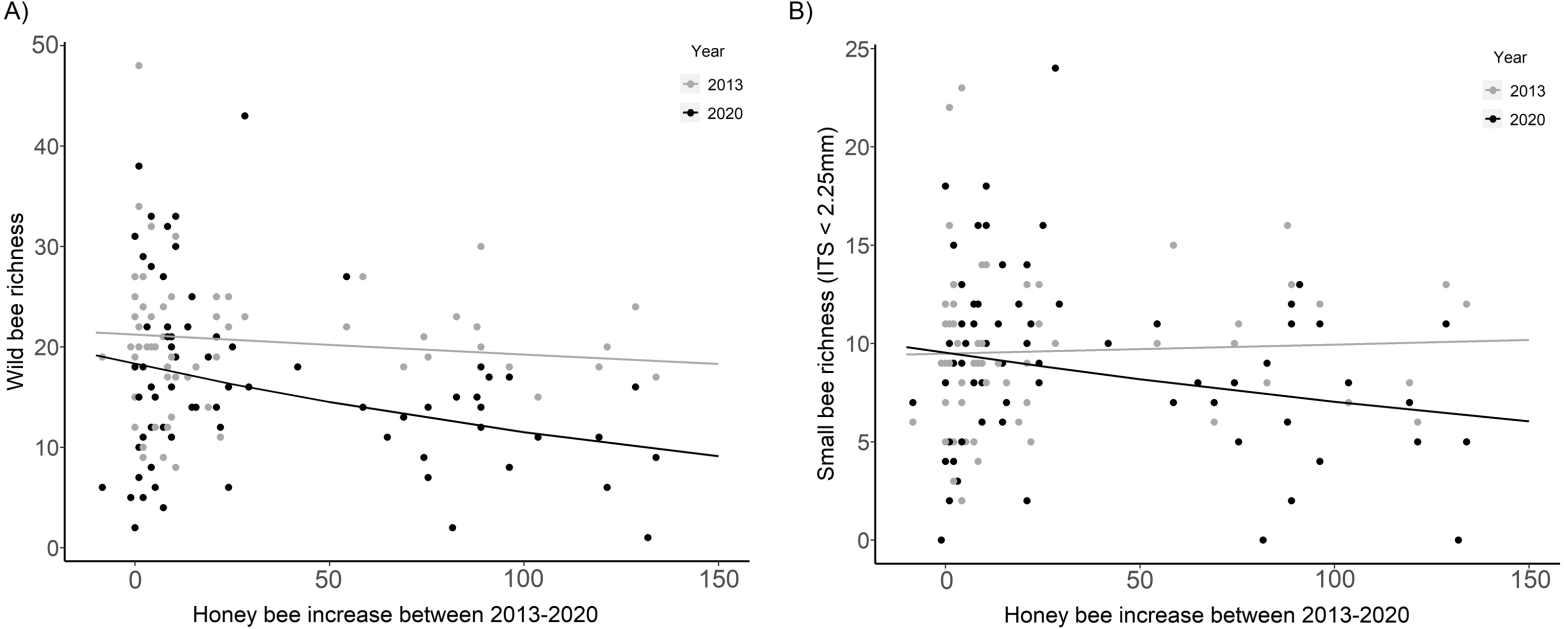
Wild bee richness and small bee richness before (2013) and after (2020) a substantial increase in urban beekeeping in Montreal, QC. (A) Richness of all wild bees; (B) richness of small wild bees in 2013 and 2020 with the increase in honey bee abundance between years. The increase in honey bee abundance was determined by taking the difference in the number of honey bees detected at each site in 2013 *versus* 2020. Honey bee presence was minimal at each site in 2013, with fewer than 20 honey bees collected in each sampling period. The solid black line is the prediction of the GLMM based on the bee community data in 2020. The solid gray line is the prediction of the GLMM based on the bee community data in 2013. Sampling period, floral richness, floral density, and the proportion of impervious surface and herbaceous greenery at 500 m buffers were included as covariates in the model.

Wild bee richness was significantly lower in 2020 than in 2013 at sites with the largest increase in honey bee abundance (HB increase × Year, *χ*_2_ = 7.99, *p* = 0.005, Fig. 4A, Table 2). The slope of the decline in wild bee richness with honey bee abundance in 2020 decreased by 78% compared to 2013 (2020: *β* = −0.23 ± 0.07, *t* =  − 3.19, *p* = 0.001; 2013: *β* = −0.05 ± 0.07, *t* =  − 0.67, *p* = 0.50). Small bee richness in 2020 was also significantly lower than in 2013 at sites with the largest increase in honey bee abundance (HB increase × Year, *χ*_2_ = 3.93, *p* = 0.047, Fig. 4B, Table 2).

**Table 2:** Results of GLMM testing of the influence of honey bee abundance on wild bee and small bee richness in 2020 vs. 2013. Data were pooled across plots at each site and sampling period in each year. Covariates included greenspace type (park, community garden, cemetery), sampling period, and the proportion of herbaceous vegetation and impervious surface within a 500 m buffer around each site. Herbaceous vegetation was the amount of green vegetation greater than 3m in height within the buffer zone was used as a proxy for floral resource availability within the foraging range of wild bees. As sampling efforts differed between years, bee richness is the rarified species richness values, standardized to equal sample coverage between years. Predictors for each model were scaled and centered on zero. *β* is the coefficients for each predictor, *CI* is the 95% confidence intervals and *p* is the significance. Significant predictors for either bee richness or abundance are in bold.

	**Wild bee richness**			**Small bee richness**		
**Predictors**	*β*	*CI*	*p*	*β*	*CI*	*p*
**(Intercept)**	3.61	2.76–4.46	**<0.001**	2.67	1.81–3.52	<0.001
**Honey bee abundance × Year**	−0.20	−0.37–0.03	**0.023**	−0.18	−0.35–0.00	**0.047**
Honey bee abundance	0.01	−0.13–0.15	0.894	0.02	−0.12–0.16	0.741
**Year**	−0.14	−0.30–0.02	0.087	0.00	−0.16–0.17	0.961
**Herbaceous veg. (%)**	−0.06	−0.45–0.33	0.751	−0.03	−0.42–0.35	0.863
Impervious surface (%)	−0.44	−0.96–0.08	0.097	−0.34	−0.86–0.18	0.202
**Greenspace type (garden)**	0.00	−0.17–0.17	0.973	0.16	−0.02–0.34	0.074
Greenspace type (park)	−0.26	−0.49–0.03	0.027	−0.00	−0.23–0.23	0.973
Sampling Period (2)	0.05	−0.15–0.26	0.600	0.05	−0.14–0.25	0.581
Sampling period (3)	0.01	−0.19–0.22	0.893	−0.06	−0.26–0.14	0.547
Sampling period (4)	−0.21	−0.42—0.00	**0.049**	−0.39	−0.61–0.18	**<0.001**
Sampling period (5)	−0.25	−0.45—0.04	**0.020**	−0.40	−0.62–0.19	**<0.001**

There was no interactive effect of honey bee increase on Shannon’s diversity (*χ*_2_ = 2.34, *p* = 0.311), Simpson’s diversity (*χ*_2_ = 1.01, *p* = 0.315) or Pielou’s evenness (*χ*_2_ = 2.56, *p* = 0.0.279) of the wild bee communities between years (Table S5). Honey bee increase had no influence on very large or large bee richness or on native or exotic bee richness between 2013 and 2020 (Table S6).

### Pollen depletion

**Figure 5:**
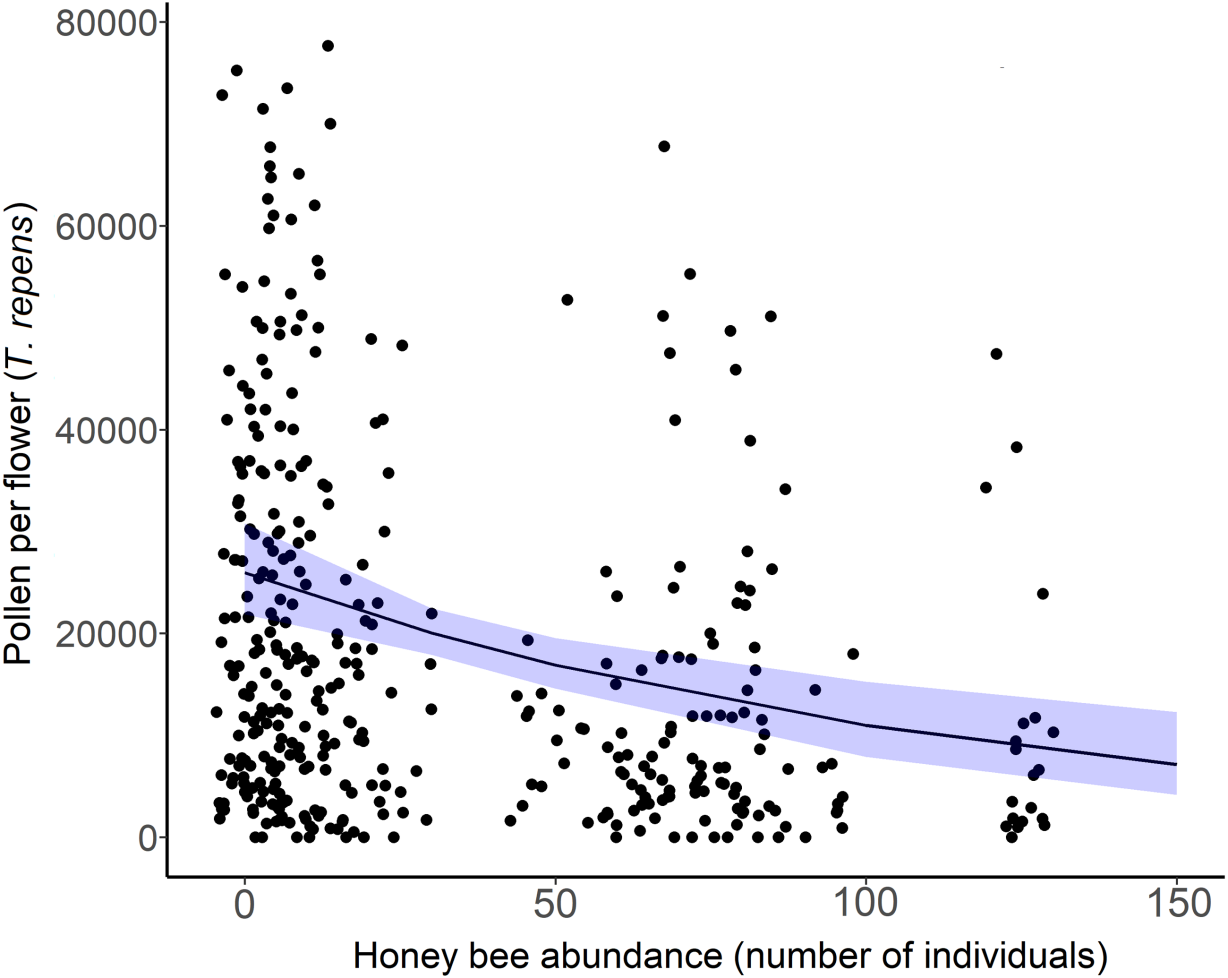
White clover (*Trifolium repens*) depletion with honey bee abundance. Each point is an individual flower, with 10 flowers measured per site over two sampling periods. Points have been jittered to improve clarity. The solid line is the prediction of the GLMM with 95% confidence intervals shown in blue. Wild bee abundance, floral richness, floral density, and sampling period were included as covariates in the model.

The amount of pollen left in white clover flowers (*T. repens*) decreased across sites as honey bee abundance increased (*β* = −0.42, ${\chi }_{2}^{1}$ = 15.36, *p* = 0.012; Fig. 5). Though pollen per flower was a continuous variable in analyses, the average pollen per flower at sites and sampling periods where more than 20 honey bees were detected (*n* = 15) was 14,483 grains per flower (*n* = 153 flowers). The average pollen per flower at sites and sampling periods that had less than 20 honey bees detected (*n* = 24) was 24,042 pollen grains per flower (*n* = 262 flowers). Honey bee abundance was the only significant predictor of pollen depletion. *Bombus* abundance did not influence clover pollen depletion (${\chi }_{2}^{1}$ = 0.1667, *p* = 0.683), however the influence of wild bee abundance on *T. repens* pollen depletion was marginally significant (*β* = −0.35, *p* = 0.074, Table 3).

## Discussion

Exploitative competition between pollinators occurs when one or more species depletes floral resources (pollen and/or nectar) to such an extent that it reduces the amount available to others (Lang & Benbow, 2013). This can either deter certain bee species from foraging in competitive flower patches, or exclude some pollinators from an ecosystem altogether. Our spatiotemporal analyses of the bee and floral communities on Montréal Island provided evidence to support our first hypothesis; that honey bee abundance negatively affects wild bee diversity through exploitative competition for urban floral resources. While accounting for differences in floral richness and density across sites, we found that wild bee richness (Fig. 2A), Shannon diversity, and the abundance of white clover pollen (*T. repens*) were negatively related to honey bee abundance in 2020 (Fig. 5). Further, most of our sites with high honey bee abundance were community gardens, which were more floral rich and dense than parks or cemeteries, and are known hotspots of bee diversity (Baldock et al., 2019; Theodorou et al., 2020). The rarefaction curves also showed that sites with higher honey bee abundance (largely gardens) were sampled more completely than sites with low honey bee abundance (Fig. S1A). Thus, we would expect the garden sites to support more bee species than park or cemetery sites, but they did not. Additionally, it is plausible that our results underestimate the negative effects of competition by honey bees on wild bees, as we missed the early season bees (*e.g.*, bees in the genus *Andrena*) due to COVID-19 pandemic-related restrictions in field site access. Early-season bees have been shown to be more vulnerable to competition by honey bees due to limited floral resource availability (Ropars et al., 2022), but late season bees may also be more vulnerable for the same reason (Hofmann, Zohner & Renner, 2019). Despite the multitude of factors expected to counter our ability to detect a signature of competition, we still detected a negative relationship between honey bee abundance and wild bee richness.

**Table 3:** Results of GLMM testing of the influence of honey bee abundance on pollen depletion in white clover (*Trifolium repens*). Covariates included, honey bee abundance, wild bee abundance, floral richness, floral density, and sampling period were included as covariates in the model. Predictors for each model were scaled and centered on zero. *β* is the coefficients for each predictor, *CI* is the 95% confidence intervals and *p* is the significance. Significant predictors of any of the three diversity metrics are in bold.

**Predictors**	**Estimate**	**CI**	***p***
(Intercept)	10.91	9.05–12.77	**<0.001**
Honey bee abundance	−0.42	−0.67—0.17	**0.012**
Wild bee abundance	−0.35	−0.74–0.03	0.074
*Bombus* abundance	−0.065	−0.225–0.095	0.683
Floral richness	−0.00	−0.41–0.40	0.982
Floral density	0.28	−0.33–0.89	0.362
Impervious surface (%)	−0.09	−1.20–1.01	0.870
Herbaceous greenery (%)	−0.70	−1.54–0.14	0.100

Although wild bee populations can fluctuate largely in space and time (Roubik & Wolda, 2001; Price et al., 2005) our analyses of the bee and floral community in 2020, and the comparison to the bee community in 2013, strongly suggests a negative relationship between high-density urban beekeeping and wild bee richness. The negative association found between wild bee richness and honey bee abundance in 2020 was not seen at these same sites in 2013 (Fig. 4A). Sites that saw the greatest increase in honey bee abundance between years had the fewest wild bee species, while honey bee abundance was found to have no influence on wild bee richness in 2013, as the population of honey bees was very low in 2013. The number of hives increased more than 12-fold between 2013 and 2020; there were 238 known hives on the island of Montréal in 2013 (McCune et al., 2020), and 2,918 known hives in 2020 (MAPAQ, G Claing, 2020, pers. comm.). However, it is important to note that differences in floral resources between years may have been underestimated here, as floral data was not collected in 2013, but in 2016. It is for this reason that we refrained from conducting any detailed analyses of the influence of the floral community between years.

Most of the bees we sampled in the Montréal wild bee community were native to our region (105 of the 120 species sampled). Our results did not detect an effect of honey bee abundance on overall native bee species richness (Table S3), but the 13 species that were absent from sites that had the highest increases in honey bee abundance since 2013, were native species (Table S4). As urban floral communities are often dominated by exotic and cultivated plants, and native bees tend to prefer native plants, exotic bees are thought to have an advantage in urban environments (Banaszak-Cibicka & Zmihorski, 2012; Frankie et al., 2019). However, generalist bees that tolerate a broad range of flowering plant species may have greater competitive ability than specialist species, regardless of native status. Indeed, we found that the abundance of two exotic generalist species (*M. rotundata, H. communis*) increased significantly with honey bee abundance, as did the abundance of three native species common to urban areas (*B. impatiens, B. griseocollis, A. virescens*). These three native species have been very successful in colonizing urban ecosystems, and *B. impatiens* is managed for pollination in greenhouses in our region, which may further augment their abundance (Colla & Packer, 2008; Sivakoff, Prajzner & Gardiner, 2018; Glaum et al., 2017). However, all *B. impatiens* sampled here were classified as wild bees, as it was not possible to differentiate between wild and managed *B. impatiens* in our study. Multiple studies have found negative relationships between *Bombus* spp. and honey bee abundance (Goulson & Sparrow, 2009; Elbgami et al., 2014; Thomson, 2016). Although, we did not find any significant negative effect of honey bee abundance on overall *Bombus* abundance, *B. impatiens* was the most abundant *Bombus* collected.

We also did not find a significant interactive influence between honey bee abundance and floral resources on wild bee richness or abundance. This result could be driven by niche partitioning in the wild bee community, whereby bees that are competing for similar resources tend to be outcompeted by honey bees, and generalist bees that can readily alternate floral resources are able to maintain their populations. *B. impatiens* in particular has been found to avoid competition with *Anthidium manicatum* (an exotic bee in our region) by foraging on floral resources outside of *A. manicatum* territory, without any apparent impact on fitness (Graham et al., 2019). Indeed, we found that the abundance and richness of flowers alone influenced both wild bee richness and abundance (but not small bee richness or abundance, see Table 1) and we detected no influence of honey bee abundance on overall wild bee abundance, or Simpson’s diversity index, which is more sensitive to declines in abundant, rather than rare, species (Morris et al., 2014). This suggests that the wild bee community currently coexisting in areas of high honey bee density in Montréal may be using alternate floral resources, whereas wild bees that compete for similar flowers to the honey bee, especially small bee species, may already have been outcompeted. Our results on pollen availability further support this reasoning. White clover pollen (*T. repens*) was depleted as honey bee abundance increased across sites, but did not decrease with *Bombus* or wild bee abundance, even though white clover is especially attractive to *Bombus* species (Larson, Kesheimer & Potter, 2014). Although honey bee consumption of white clover pollen decreases the amount available for other species (a prerequisite for exploitative competition), it may have increased the availability of alternate floral resources for other bees, if honey bees preferentially foraged on *T.repens*. Further, *T. repens* was one of the most abundant floral resources across sites (Table S8) and honey bees are known to stay constant to abundant and rewarding floral resources, including *T. repens* (Free, 1963; Kanduth et al., 2021).

Competitive interactions between bee species are complex and are not likely to be consistent among bee communities, traits, habitats, regions, and landscapes. The relative competitive abilities of bee species will vary diurnally, by year, with temperature, habitat, floral resource type and availability, morphologies, life histories and interactions among these variables (Corbet et al., 1995; Prendergast, Dixon & Bateman, 2021; Su et al., 2022). In our study, small bee species (ITS <2.25 mm) were influenced by the presence of managed honey bees. The analyses of the small wild bee community (57% of all wild bees sampled) showed a decline in richness with honey bee abundance across both sites and years (Figs. 2B and 4B), not seen in the large bee community. Although small bees require fewer floral resources than large bees, they do not have the ability to forage long distances in times of increased competition (Zurbuchen et al., 2010). While large bees (especially those in the genus *Bombus*) require more sustenance, they can often forage earlier in the day and in colder conditions than most smaller bees (Heinrich, 1972; Peters et al., 2016). The long tongues of most large bees also allow them to forage on flowers with deep corollas, whose nectar is inaccessible to many small bees. Short-tongued bees have proboscis lengths of 0.5–3 mm, and medium-tongued bees have lengths of 3.5−5.5 mm (O’Toole & Raw, 1991); honey bees can extend their proboscis about 7 mm. Thus, large bees and honey bees are likely to have a competitive advantage in colder climates, and in patches of deep flowers. The floral communities analyzed in this study were largely dominated by flowers with deep corollas. Seventy-three percent of the flower species sampled in this study had corollas greater than 5.5 mm, and 90% had corollas greater than 3.0 mm (data not shown). Although some small bees can access tubular flowers by crawling inside, this requires more effort and may have increased their susceptibility to competition by larger species in the floral communities studied here.

Despite the natural spatiotemporal variation in bee community composition and the complexity of competitive interactions among bee species, we detected a significantly negative relationship between honeybee abundance and wild bee richness. However, this study is correlative by design, and provides only indirect evidence of exploitative competition. Negative correlations between honey bee populations and wild bee diversity cannot be interpreted as direct evidence for competitive exclusion. To compliment field studies, more experimental investigations of the fitness impacts of honey bees on wild bee species over multiple years are needed to further our understanding of interspecies competition, and provide reliable estimates of the carrying capacity of cities for honey bees (Wojcik et al., 2018). However, the results presented here warrant further research and a cautionary approach to urban beekeeping. Montréal’s urban bee community is largely dominated by honey bees, which comprised almost 40% of all bees detected in this study. In addition to increasing the competitive pressure between and among bee species, keeping managed bees in such high densities poses a health risk to both managed and wild bees though increased pest and pathogen transfer (Graystock et al., 2016; Nanetti, Bortolotti & Cilia, 2021; Requier et al., 2017; Tehel, Brown & Paxton, 2016). Hive densities on the order of 6/km^2^ in Paris (Ropars et al., 2019) were negatively correlated with wild bee foraging activity, and above 10/km^2^ in Slovenia increased the prevalence of viruses in both bumble bees and honey bees (Ocepek et al., 2021). If the approximately 3,000 hives in Montréal were distributed equally across the island, hive densities would be on the order of 6.5/km^2^, though in reality this distribution is not equal. Although honey bee colony carrying capacity would vary by city and floral resource availability, precautionary recommended colony densities are on the order of 3−3.5/km^2^ to reduce negative interspecies interactions (Steffan-Dewenter & Tscharntke, 2000; Torné-Noguera et al., 2016).

Our ability to make recommendations on the honey bee colony carrying capacity of Montréal’s greenspaces was limited by unknown hive densities and locations. The lack of city beekeeping registries, coupled with the large foraging ranges of honey bees are substantial impediments to research on interspecies competition. This makes effective study design especially challenging, as it is almost impossible to locate control sites that lack honey bees. As a result, our honey bee abundance data was based on the number of honey bees detected during 10-minute aerial netting periods at the site level, with no means to translate this data to colony densities at local levels. *A priori* site selection was informed by hive data provided on large, often regional scales, and on rough estimates of hive densities gleaned from maps provided by beekeepers who were willing to provide information on their apiary locations. To facilitate effective field studies and mitigate the formation of high-density colony sites, it is imperative for cities to maintain a registry of beekeepers with hive locations.

Beekeeping can be considered a form of livestock management, but unlike most livestock animals, a beekeeper is not required to provide sustenance for their bees. Honey bees are highly mobile organisms that are free to roam and exploit floral resources in their environment. As such, the introduction of honey bee colonies in cities usually does not coincide with an increase in the pollen and nectar required to sustain the wild bee community in addition to burgeoning honey bee populations (Casanelles-Abella & Moretti, 2022). Given that competition between bee species increases when floral resources are limited, the potential for negative interactions between honey bees and wild bees could be reduced by increasing pollen and nectar sources in tandem with new colonies. However, the addition of floral resources to the urban landscape does not address a negative influence of pathogen prevalence and spillover among and between bee species in high-density bee communities. Our study focused largely on wild bee species (with both native and exotic status), which declined with honey bee abundance. We found no influence of honey bee abundance specifically on bees native to our region, but our results showed that native bee species richness and abundance was positively influenced by floral richness and density. To support our native bees and encourage ecologically responsible urban beekeeping, introductions of honey bee colonies could be accompanied by deliberate planting of flowers known to provide abundant pollinator resources, especially those preferred by more vulnerable or specialist bee species. Additionally, the creation of more evidence-based frameworks to regulate urban beekeeping operations are paramount to develop sustainable beekeeping operations while protecting wild pollinator diversity in cities.

##  Supplemental Information

10.7717/peerj.14699/supp-1 Supplemental Information 1 Supplementary Figure and Tables

10.7717/peerj.14699/supp-2 File S1 Species abundance matrix for each site in 2013 and 2020

10.7717/peerj.14699/supp-3 File S2 Wild bee species richness and abundance with bee traits and covariates used for statistical analyses

10.7717/peerj.14699/supp-4 File S3 Wild bee species richness and abundance with bee traits and covariates used for statistical analyses for the year 2013 and 2020

10.7717/peerj.14699/supp-5 Supplemental Information 5 Metadata for each of the three datasets Describes each variable in the accompanying datasets.

## Additional Information and Declarations

### Field Study Permissions

The following information was supplied relating to field study approvals (*i.e.*, approving body and any reference numbers): Permission to access to field sites was given by The City of Montreal - Direction de la Gestion des Parcs et de la Biodiversité, and by community garden and cemetery managers.

### Data Availability

The following information was supplied regarding data availability: The raw data are available in the Supplemental Files.
