# Supplementary material for: Decline in wild bee species richness associated with honey bee (Apis mellifera L.) abundance in an urban ecosystem
Source: PeerJ. 2023 Feb 3;11:e14699. doi: 10.7717/peerj.14699 (PMC9901307; doi:10.7717/peerj.14699)
Supplement: Supplemental Information 1 [file peerj-11-14699-s001.docx]

# Supplementary Material


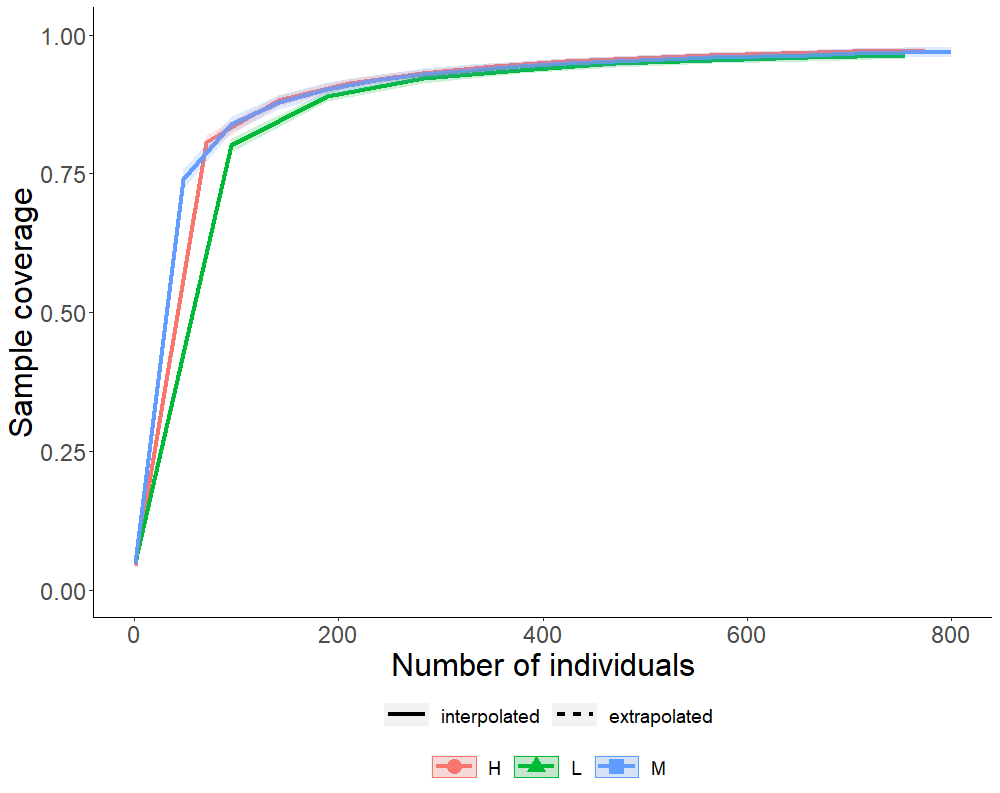

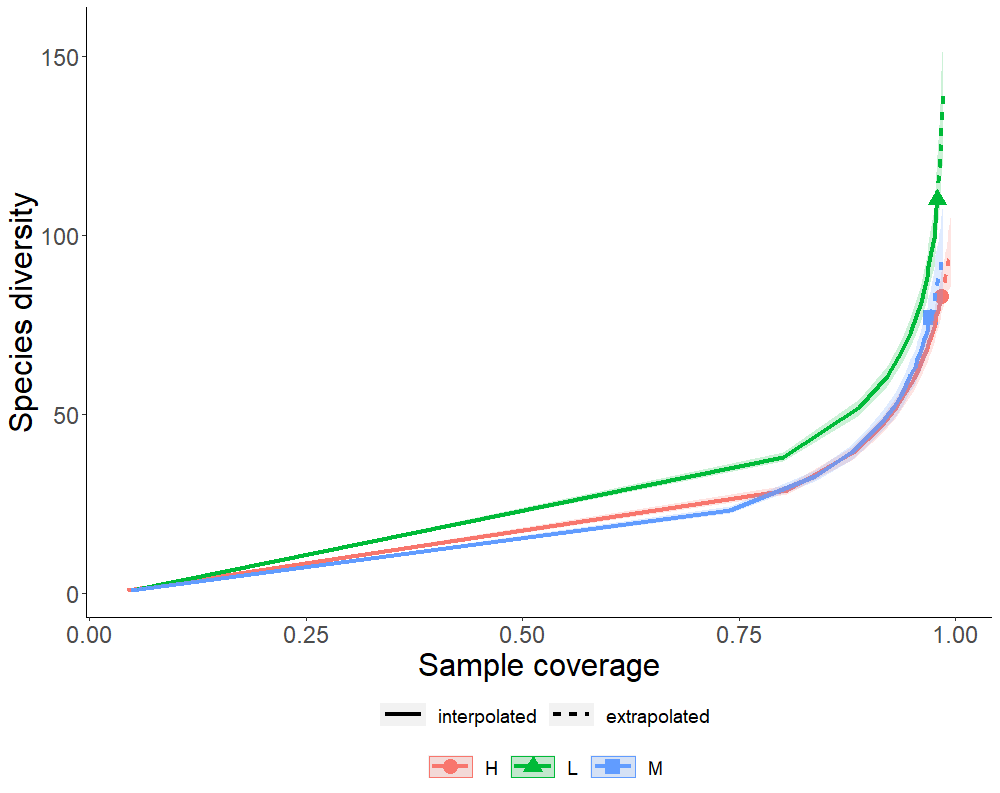


## *Figure S.1: Coverage-base rarefaction curves and species richness with coverage. In a) the solid lines represent the interpolated percent sample coverage and in b) the solid line represents the interpolated sample-based rarefaction curve; the dotted section are the extrapolated sections of the curves.*

## *Table S.1: Average Impervious surface and green area with honey bee abundance and greenspace type. Honey bee abundance indicates the average number of honey bees detected during sampling periods. Herbaceous vegetation was the amount of green vegetation of > 3m in height within a buffer zone of 500m, centered on the study sites, and was used as a proxy for floral resource availability within the foraging range of wild bees. Impervious surface was calculated in the same buffer zone, and was a proxy for habitat availability. Floral richness and density were measured locally at each site.*

|  | **HB abundance (site\|sampling period)** | | | **Greenspace type (site)** | | |
| --- | --- | --- | --- | --- | --- | --- |
|  | **> 20 HB**  **(n=39)** | **20-40 HB**  **(n=13)** | **> 40 HB**  **(n = 22)** | **Garden**  **(n=9)** | **Park**  **(n=2)** | **Cemetery**  **(n=4)** |
| **Herbaceous greenery**  Mean ± SD (%) | 27.70 ± 8.52 | 24.18 ± 11.07 | 18.68 ± 12.31 | 29.73 ± 19.09 | 27.33 ± 3.06 | 25.67 ± 11.02 |
| **Impervious surface**  Mean ± SD (%) | 0.54 ± 0.15 | 0.59 ± 0.18 | 0.65 ± 0.19 | 62.48 ± 17.21 | 44.75 ± 8.70 | 57.00 ± 3.06 |
| **Floral richness** | 9.53 ± 6.07 | 12.30 ± 6.67 | 18.3 ± 6.78 | 17.08 ± 5.84 | 5.5 ± 2.33 | 6.06 ± 2.93 |
| **Floral density** | 0.072 ± 0.035 | 0.086 ± 0.034 | 0.087 ± 0.026 | 0.08 ± 0.03 | 0.09 ± 0.02 | 0.06 ± 0.04 |

## *Table S.2: Results of GLMM testing of the influence of honey bee abundance on wild bee diversity metrics. Data were pooled across plots at each site and sampling period (N = 8 plots per site, 15 sites, 5 sampling periods). Shannon-H is the Shannon-Weiner diversity index, Simpson-D is Simpson’s diversity index and Pilou’s J is Pielous evenness index (Shannon-H/ln(species)). Covariates included, floral richness, floral density, greenspace type (park, community garden, cemetery), sampling period, and the proportion of herbaceous vegetation and impervious surface within a 500 m buffer around each site. Floral richness and density were measured locally at each site, and herbaceous vegetation was the amount of green vegetation of >3m in height within the buffer zone, and was used as a proxy for floral resource availability within the foraging range of wild bees. Predictors for each model were scaled and centered on zero. β is the coefficients for each predictor, Cl is the 95% confidence intervals and p is the significance. Significant predictors of any of the three diversity metrics are in bold.*

|  | **Diversity**  **(Shannon-H)** | | | **Diversity**  **(Simpson-D)** | | | **Evenness**  **(Pielou’s J)** | | |
| --- | --- | --- | --- | --- | --- | --- | --- | --- | --- |
| Predictors | *β* | CI | p | *β* | *CI* | *p* | β | Cl | *p* |
| (Intercept) | 1.51 | -1.13 – 4.16 | 0.262 | 0.66 | -0.00 – 1.32 | 0.051 | 1.18 | 0.78 – 1.57 | <0.001 |
| **Honey bee abundance** | -0.27 | -0.54 – -0.01 | **0.045** | -0.02 | -0.08 – 0.05 | 0.656 | -0.01 | -0.05 – 0.04 | 0.730 |
| **Floral richness** | 0.55 | 0.01 – 1.08 | **0.044** | 0.16 | 0.02 – 0.30 | **0.023** | -0.06 | -0.15 – 0.02 | 0.147 |
| **Floral density** | -0.01 | -0.42 – 0.41 | 0.975 | -0.09 | -0.15 – 0.07 | **0.040** | -0.10 | -0.18 – -0.01 | **0.028** |
| Herbaceous veg. (%) | 0.14 | -0.93 – 1.20 | 0.801 | 0.09 | -0.18 – 0.35 | 0.523 | -0.02 | -0.17 – 0.13 | 0.819 |
| Impervious surface (%) | 0.02 | -1.53 – 1.56 | 0.983 | 0.05 | -0.33 – 0.43 | 0.787 | -0.11 | -0.32 – 0.11 | 0.332 |
| Greenspace type (garden) | 0.35 | -0.29 – 0.99 | 0.282 | -0.00 | -0.16 – 0.16 | 0.971 | 0.03 | -0.07 – 0.12 | 0.571 |
| Greenspace type (park) | 0.41 | -0.26 – 1.08 | 0.233 | 0.08 | -0.09 – 0.24 | 0.362 | -0.07 | -0.17 – 0.02 | 0.140 |
| Sampling period (2) | 0.29 | -0.60 – 1.19 | 0.525 | 0.04 | -0.20 – 0.29 | 0.720 | -0.00 | -0.20 – 0.19 | 0.974 |
| Sampling period (3) | 0.10 | -0.80 – 1.00 | 0.826 | -0.01 | -0.25 – 0.23 | 0.928 | -0.04 | -0.24 – 0.15 | 0.667 |
| Sampling period (4) | -0.24 | -1.13 – 0.65 | 0.596 | -0.06 | -0.31 – 0.18 | 0.602 | -0.06 | -0.26 – 0.14 | 0.552 |
| Sampling period (5) | -0.36 | -1.26 – 0.54 | 0.432 | -0.09 | -0.34 – 0.15 | 0.461 | -0.08 | -0.28 – 0.11 | 0.402 |

## *Table S.3. Results of GLMM testing of the influence of honey bee abundance on wild bee community traits in 2020. Data were pooled across plots at each site and sampling period (N = 8 plots per site, 15 sites, 5 sampling periods). Covariates included, floral richness, floral density, greenspace type (park, community garden, cemetery), sampling period, and the proportion of herbaceous vegetation and impervious surface within a 500 m buffer around each site. Bombus models used a 1500 m buffer to account for the larger foraging range of these bees. Floral richness and density were measured locally at each site, and herbaceous vegetation was the amount of green vegetation of >3m in height within the buffer zone, and was used as a proxy for floral resource availability within the foraging range of wild bees. Predictors for each model were scaled and centered on zero. β is the coefficients for each predictor, Cl is the 95% confidence intervals and p is the significance. Significant predictors of any of the three diversity metrics are in bold.*

| Response trait | **Estimate** | **CL** | **HB Abundance**  χ^2^  *p* | |
| --- | --- | --- | --- | --- |
| Native species richness | 0.09 | -0.09 – 0.27 | 0.87 | 0.350 |
| Native abundance | 0.06 | -0.25 – 0.38 | 0.14 | 0.707 |
| Exotic species | 0.02 | -0.20 – 0.24 | 0.03 | 0.871 |
| Exotic abundance | -0.10 | -0.45 – 0.25 | 0.32 | 0.573 |
| **Small bee richness** | -0.31 | -0.59 - -0.03 | 4.76 | **0.029** |
| **Small bee abundance** | -0.45 | -0.82 - -0.08 | 3.94 | **0.019** |
| Large bee richness | 0.15 | -0.04 – 0.35 | 2.30 | 0.129 |
| Large bee abundance | -0.06 | -0.24 – 0.13 | 0.36 | 0.548 |
| *Bombus* richness | 0.21 | -0.13 – 0.55 | 1.46 | 0.227 |
| *Bombus* abundance | 0.12 | -0.16 – 0.39 | 0.72 | 0.397 |

## *Table S.4.* *Wild bee species absent from sites that saw the highest increase in honey bee abundance since 2013. First and second columns indicate the number of each species at these high honey bee abundance sites (sites where more than 20 honey bees were detected in sampling periods). The third and fourth columns show the abundance of the same wild bee species at sites that had fewer honey bees in 2020 (with 20 or fewer honey bees detected during sampling periods).*

|  | Species | Sites with > 20 HB in | Abundance in 2013 | Sites with ≤ 20 HB 2020 | Abundance in 2013 |
| --- | --- | --- | --- | --- | --- |
| **1** | *Andrena cratagei* | 0 | 14 | 2 | 15 |
| **2** | *Augochlora aurata* | 0 | 6 | 0 | 196 |
| **3** | *Andrena wheeleri* | 0 | 1 | 7 | 10 |
| **4** | *Andrena nassoni* | 0 | 1 | 1 | 1 |
| **5** | *Augochlora pura* | 0 | 1 | 19 | 12 |
| **6** | *Bombus citrinus* | 0 | 3 | 3 | 3 |
| **7** | *Bombus vagans* | 0 | 1 | 2 | 4 |
| **8** | *Coelioxys porterae* | 0 | 7 | 1 | 2 |
| **9** | *Hoplitis spoliata* | 0 | 4 | 15 | 7 |
| **10** | *Lasioglossum macoupinense* | 0 | 1 | 2 | 9 |
| **11** | *Lasioglossum planatum* | 0 | 3 | 1 | 1 |
| **12** | *Melissodes druriellus* | 0 | 1 | 2 | 8 |
| **13** | *Melissodes illata* | 0 | 28 | 1 | 6 |

*Table S.5. Results of GLMM testing of the influence of honey bee increase between 2013 and 2020 on wild bee diversity metrics.* *Data were pooled across plots at each site and sampling period, N = 15 sites (2020), N = 13 sites (2013), 5 sampling periods. Shannon-H is the Shannon-Weiner diversity index, Simpson-D is Simpson’s diversity index and Pilou’s J is Pielous evenness index (Shannon-H/ln(species)) Covariates included greenspace type (park, community garden, cemetery), sampling period, and the proportion of herbaceous vegetation and impervious surface within a 500 m buffer around each site. Herbaceous vegetation was the amount of green vegetation of >3m in height within the buffer zone, and was used as a proxy for floral resource availability within the foraging range of wild bees. Local floral richness and density were not measured in 2013, but were in 2016, thus the floral richness and density were. Predictors for each model were scaled and centered on zero. Significant predictors of any of the three diversity metrics are in bold.*

|  | **Diversity**  **(Shannon-H)** | | | **Diversity**  **(Simpson-D)** | | | **Evenness**  **(Pielou’s J)** | | |
| --- | --- | --- | --- | --- | --- | --- | --- | --- | --- |
| *Predictors* | *β* | *CI* | *p* | *β* | *CI* | *p* | *β* | *Cl* | *p* |
| (Intercept) | 2.99 | 1.68 – 4.31 | **<0.001** | 0.84 | 0.60 – 1.08 | <0.001 | 0.66 | 0.44 – 0.87 | **<0.001** |
| Honey bee abundance x Year | 0.02 | -0.18 – 0.21 | 0.869 | -0.02 | -0.06 – 0.02 | 0.339 | -0.03 | -0.07 – 0.01 | 0.191 |
| Honey bee abundance | 0.01 | -0.18 – 0.20 | 0.943 | 0.01 | -0.03 – 0.06 | 0.523 | 0.01 | -0.03 – 0.04 | 0.766 |
| Year (2020) | -0.06 | -0.25 – 0.13 | 0.547 | 0.01 | -0.03 – 0.06 | 0.523 | 0.07 | 0.03 – 0.11 | **0.001** |
| Herbaceous veg. (%) | -0.13 | -0.73 – 0.46 | 0.663 | 0.03 | -0.08 – 0.14 | 0.546 | 0.10 | -0.00 – 0.19 | 0.051 |
| Impervious surface | -0.47 | -1.28 – 0.33 | 0.249 | -0.02 | -0.17 – 0.12 | 0.776 | 0.10 | -0.03 – 0.23 | 0.141 |
| Greenspace Type[Garden] | 0.19 | -0.08 – 0.46 | 0.167 | 0.03 | -0.02 – 0.07 | 0.319 | -0.01 | -0.05 – 0.04 | 0.814 |
| Greenspace Type [Park] | -0.01 | -0.36 – 0.35 | 0.972 | 0.00 | -0.06 – 0.07 | 0.932 | -0.00 | -0.06 – 0.05 | 0.876 |
| Sampling period [2] | -0.10 | -0.34 – 0.14 | 0.420 | -0.02 | -0.07 – 0.04 | 0.569 | -0.02 | -0.07 – 0.04 | 0.554 |
| Sampling period [3] | -0.18 | -0.42 – 0.06 | 0.140 | -0.03 | -0.08 – 0.03 | 0.327 | -0.01 | -0.06 – 0.04 | 0.743 |
| Sampling period [4] | -0.63 | -0.87 – -0.39 | **<0.001** | -0.11 | -0.16 – -0.06 | <0.001 | -0.04 | -0.09 – 0.01 | 0.132 |
| Sampling period [5] | -0.19 | -0.44 – 0.05 | 0.121 | -0.03 | -0.08 – 0.02 | 0.275 | -0.02 | -0.08 – 0.03 | 0.379 |

## Table S.6. The influence of honey bee abundance on richness in bee community trait groups in 2020 vs. 2013

| Response trait | **Estimate** | CL | HB increase × Year  χ^2^  *p* | |
| --- | --- | --- | --- | --- |
| Native species richness | 0.05 | -0.11 – 0.22 | 0.39 | 0.531 |
| Exotic species richness | -0.08 | -0.29 – 0.12 | 0.67 | 0.412 |
| **Small bee richness** | - 0.18 | -0.35 – 0.00 | 3.95 | **0.047** |
| Large bee richness | 0.18 | -0.01 – 0.36 | 3.60 | 0.058 |
| Bombus richness | 0.22 | -0.06 – 0.50 | 2.34 | 0.126 |

## Table S.7: Species list, with the number of individual specimens observed or collected. Letter and number designations for species epithet indicates morphospecies designation.

| Species | Individuals |
| --- | --- |
| *Apis mellifera* Linnaeus, 1958 | 2291 |
| *Ceratina calcarata* Robertson, 1900 | 311 |
| *Agapostemon virescens* Fabricius, 1775 | 260 |
| *Hylaeus communis* Nylander, 1852 | 238 |
| *Hylaeus hyalinatus* Smith, 1842 | 204 |
| *Lasioglossum laevissimum* Smith, 1853 | 192 |
| *Bombus impatiens* Cresson, 1863 | 186 |
| *Lasioglossum imitatum* Smith, 1853 | 169 |
| *Halictus ligatus* Say, 1837 | 141 |
| *Bombus griseocollis* De Geer, 1773 | 127 |
| *Megachile rotundata* Fabricius, 1787 | 123 |
| *Ceratina mikmaqi* Rehan and Sheffield, 2011 | 117 |
| *Hylaeus punctatus* Brullé, 1832 | 113 |
| *Hoplitis producta* Cresson, 1864 | 106 |
| *Halictus rubicundus* Christ, 1791 | 101 |
| *Hylaeus modestus* Say, 1837 | 86 |
| *Ceratina dupla* Say, 1837 | 85 |
| *Lasioglossum lineatulum* Crawford, 1906 | 77 |
| *Anthidium florentinum* Fabricius, 1775 | 74 |
| *Hylaeus mesillae* Cockerell, 1896 | 74 |
| *Halictus confusus* Smith, 1853 | 71 |
| *Lasioglossum cinctipes* Provancher, 1888 | 65 |
| *Chelostoma rapunculi* Lepeletier, 1841 | 56 |
| *Lasioglossum leucozonium* Schrank, 1781 | 49 |
| *Hylaeus affinis* Smith, 1853 | 46 |
| *Megachile mendica* Cresson, 1878 | 42 |
| *Megachile texana* Cresson, 1878 | 39 |
| *Lasioglossum sagax* Sandhouse, 1924 | 38 |
| *Lasioglossum tegulare* Robertson, 1890 | 34 |
| *Bombus rufocinctus* Cresson, 1863 | 30 |
| *Anthidium manicatum* Linnaeus, 1758 | 28 |
| *Melissodes subillatus* LaBerge, 1961 | 28 |
| *Bombus bimaculatus* Cresson, 1863 | 27 |
| *Lasioglossum coriaceum* Smith, 1853 | 27 |
| *Lasioglossum zonulum* Smith, 1848 | 25 |
| *Lasioglossum ephialtum* Gibbs, 2010 | 24 |
| *Lasioglossum pilosum* Smith, 1853 | 24 |
| *Andrena wilkella* Kirby, 1802 | 23 |
| *Hylaeus leptocephalus* Morawitz, 1871 | 22 |
| *Augochlora pura* Say, 1837 | 21 |
| *Anthidium oblongatum* Illiger, 1806 | 20 |
| *Augochlorella aurata* Smith, 1853 | 20 |
| *Andrena commoda* Smith, 1879 | 17 |
| *Chelostoma campanularum* Kirby, 1802 | 17 |
| *Lasioglossum oenotherae* Stevens, 1920 | 17 |
| *Hoplitis spoliata* Provancher, 1888 | 15 |
| *Hylaeus annulatus* Linnaeus, 1758 | 15 |
| *Megachile campanulae* Robertson, 1903 | 15 |
| *Megachile brevis* Say, 1837 | 14 |
| *Coelioxys sayi* Robertson, 1897 | 12 |
| *Megachile lapponica* Thomson, 1872 | 12 |
| *Melissodes desponsus* Smith, 1854 | 11 |
| Megachile A1 | 10 |
| *Peponapis pruinosa* Say, 1837 | 10 |
| *Anthophora terminalis* Cresson, 1869 | 8 |
| Lasioglossum A1 | 8 |
| *Lasioglossum pectorale* Smith, 1853 | 8 |
| *Lasioglossum quebecense* Crawford, 1907 | 8 |
| *Andrena wheeleri* Graenicher, 1904 | 7 |
| *Hoplitis pilosifrons* Cresson, 1864 | 7 |
| *Lasioglossum cressonii* Robertson, 1890 | 7 |
| *Lasioglossum versatum* Robertson, 1902 | 7 |
| *Megachile relativa* Cresson, 1878 | 7 |
| *Lasioglossum hitchensi* Gibbs, 2012 | 6 |
| *Lasioglossum perpunctatum* Ellis, 1913 | 6 |
| *Andrena nivalis* Smith, 1853 | 4 |
| *Coelioxys octodentatus* Say, 1824 | 4 |
| Hoplitis A1 | 4 |
| *Lasioglossum admirandum* Sandhouse, 1924 | 4 |
| *Lasioglossum foxii* Robertson, 1895 | 4 |
| *Lasioglossum versans* Lovell, 1905 | 4 |
| *Osmia albiventris* Cresson, 1864 | 4 |
| *Agapostemon sericeus* Forster, 1771 | 3 |
| *Andrena cressonii* Robertson, 1891 | 3 |
| *Bombus citrinus* Smith, 1854 | 3 |
| *Bombus fervidus* Fabricius, 1798 | 3 |
| *Lasioglossum albipenne* Robertson, 1890 | 3 |
| *Lasioglossum smilacinae* Robertson, 1897 | 3 |
| *Lasioglossum zephyrum* Smith, 1853 | 3 |
| *Megachile centuncularis* Linnaeus, 1758 | 3 |
| Megachile A2 | 3 |
| *Megachile frigida* Smith, 1853 | 3 |
| *Agapostemon texanus* Cresson, 1872 | 2 |
| *Andrena cratagei* Robertson, 1893 | 2 |
| *Andrena w-scripta* Viereck, 1904 | 2 |
| *Bombus terricola* Kirby, 1837 | 2 |
| *Bombus vagans* Smith, 1854 | 2 |
| *Coelioxys rufitarsis* Smith, 1854 | 2 |
| *Lasioglossum macoupinense* Robertson, 1895 | 2 |
| *Melissodes druriellus* Kirby, 1802 | 2 |
| Osmia A1 | 2 |
| *Sphecodes dichrous* Smith, 1853 | 2 |
| *Andrena geranii* Robertson, 1891 | 1 |
| *Andrena imitatrix* Cresson, 1872 | 1 |
| *Andrena nasonii* Robertson, 1895 | 1 |
| *Andrena robertsonii* Dalla Torre, 1896 | 1 |
| *Calliopsis andreniformis* Smith, 1853 | 1 |
| *Coelioxys porterae* Cockerell, 1900 | 1 |
| *Hoplitis anthocopoides* Schenck, 1853 | 1 |
| *Lasioglossum bruneri* Crawford, 1902 | 1 |
| *Lasioglossum heterognathum* Mitchell, 1960 | 1 |
| *Lasioglossum leucocomum* Lovell, 1908 | 1 |
| *Lasioglossum nigroviride* Graenicher, 1911 | 1 |
| *Lasioglossum oblongum* Lovell, 1905 | 1 |
| *Lasioglossum planatum* Lovell, 1905 | 1 |
| Lasioglossum A2 | 1 |
| *Lasioglossum subversans* Mitchell, 1960 | 1 |
| Lasioglossum A3 | 1 |
| *Lasioglossum viridatum* Lovell, 1905 | 1 |
| *Macropis nuda* Provancher, 1882 | 1 |
| *Megachile latimanus* Say, 1823 | 1 |
| *Megachile melanophaea* Smith, 1853 | 1 |
| *Megachile montivaga* Cresson, 1878 | 1 |
| *Megachile pugnata* Say, 1837 | 1 |
| Melissodes A1 | 1 |
| *Melissodes illata* Lovell and Cockerell, 1906 | 1 |
| *Melissodes trinodis* Robertson, 1901 | 1 |
| *Osmia pumila* Cresson, 1864 | 1 |
| *Osmia sandhouseae* Mitchell, 1927 | 1 |
| *Sphecodes dichrous Smith,* 1853 | 1 |
| Stelis A1 | 1 |
| Total | 120 species  6185 individuals (3894 wild bees) |

## Table S.8: Flowering plant species recorded at across all sites with the number of individuals counted and the percent cover averaged over eight, 2 m x 2 m quadrats centered on the pan trap locations.

| Species | Individuals | % cover |
| --- | --- | --- |
| *Erigeron strigosus* | 50 | 11.53 |
| *Calibrachoa parviflora* | 47 | 9.585106 |
| *Tagetes patula* | 46 | 6.728261 |
| *Viola tricolor* | 35 | 5.885714 |
| *Trifolium repens* | 33 | 9.166667 |
| *Sonchus arvensis* | 32 | 10.07813 |
| *Cosmos sulphureus* | 31 | 9.903226 |
| *Thymus x citriodorus* | 30 | 11.86667 |
| *Achillea millefolium* | 29 | 8.982759 |
| *Solanum melongena* | 27 | 10.61111 |
| *Levisticum officinale* | 26 | 10.76923 |
| *Erigeron annuus* | 25 | 11.94 |
| *Rudbeckia laciniata* | 24 | 11.5625 |
| *Hysop officinalis* | 23 | 4.065217 |
| *Osteospermum sp.* | 23 | 8.152174 |
| *Centaurea jacea* | 22 | 17.81818 |
| *Physalis philadelphica* | 21 | 12.2381 |
| *Tropaeolum majus* | 19 | 12.10526 |
| *Dasiphora fruticosa* | 17 | 4.411765 |
| *Coreopsis grandiflora* | 16 | 7.03125 |
| *Cucurbita pepo* | 16 | 9.633333 |
| *Anethum graveolens* | 15 | 6.666667 |
| *Rosa moyesii* | 15 | 8.333333 |
| *Valeriana officinalis* | 15 | 4.6 |
| *Allium cepa* | 14 | 7.392857 |
| *Lunaria annua* | 14 | 7.714286 |
| *Salvia officinalis* | 14 | 5.821429 |
| *Solanum lycopersicum* | 14 | 3.392857 |
| *Begonia evansiana* | 13 | 12.30769 |
| *Cosmos bipinnatus* | 13 | 13.84615 |
| *Hydrangea paniculata* | 13 | 24.66667 |
| *Taraxacum officinale* | 13 | 7.692308 |
| *Hemerocallis lilioasphodelus* | 12 | 10.70833 |
| *Brassica rapa* | 11 | 9.318182 |
| *Hesparis matronalis* | 11 | 7.954545 |
| *Medicago lupulina* | 11 | 9.863636 |
| *Pelargonium x hortorum* | 11 | 12.59091 |
| *Phacelia tanacetifolia* | 11 | 4.454545 |
| *Viola x wittrockiana* | 11 | 11.27273 |
| *Helianthus annuus* | 10 | 9.95 |
| *Momordica charantia* | 10 | 7.1 |
| *Tanacetum vulgare* | 10 | 11.75 |
| *Antirrhinum majus* | 9 | 5.111111 |
| *Galinsoga quadriradiata* | 9 | 5.555556 |
| *Rudbeckia triloba* | 9 | 14.44444 |
| *Verbena bonariensis* | 9 | 5.166667 |
| *Campanula rapunculoides* | 8 | 5.1875 |
| *Lavandula dentata* | 8 | 12.6875 |
| *Phlox drummondii* | 8 | 14.125 |
| *Phlox paniculata* | 8 | 16.25 |
| *Trifolium pratense* | 8 | 2.5 |
| *Delphinium elatum* | 7 | 3.214286 |
| *Lactuca sativa* | 7 | 6.785714 |
| *Lilium bulbiferum* | 7 | 9.642857 |
| *Paeonia sp.* | 7 | 3.928571 |
| *Ageratum houstonianum* | 6 | 5.666667 |
| *Chrysanthemum leucanthemum* | 6 | 3.333333 |
| *Hibiscus moscheutos* | 6 | 6.666667 |
| *Hyacinthoides hispanica* | 6 | 6.083333 |
| *Lagenaria siceraria* | 6 | 5.666667 |
| *Pastinaca sativa* | 6 | 3.75 |
| *Phlox maculata* | 6 | 8.666667 |
| *Prunella vulgaris* | 6 | 2.916667 |
| *Raphanus raphanistrum* | 6 | 4.833333 |
| *Tanacetum parthenium* | 6 | 7.666667 |
| *Campanula latifolia* | 5 | 4.9 |
| *Celosia argentea* | 5 | 4.6 |
| *Daucus carota* | 5 | 12 |
| *Gaillardia x grandiflora* | 5 | 27 |
| *Inconnu* | 5 | 9.6 |
| *Lantana camara* | 5 | 5 |
| *Malcomia maritima* | 5 | 11.5 |
| *Perilla frutescens* | 5 | 3.3 |
| *Pisum sativum* | 5 | 6.5 |
| *Rosa cinnamomea* | 5 | 4.4 |
| *Thymus vulgaris* | 5 | 5.1 |
| *Agastache foeniculum* | 4 | 5 |
| *Aquilegia vulgaris* | 4 | 6.12 |
| *Asclepias tuberosa* | 4 | 8.125 |
| *Crepis capillaris* | 4 | 22.5 |
| *Impatiens walleriana* | 4 | 7.375 |
| *Lotus corniculatus* | 4 | 8.125 |
| *Malva moschata* | 4 | 10.5 |
| *Medicago sativa* | 4 | 38.5 |
| *Ocimum minimum* | 4 | 2.875 |
| *Rosa chinensis* | 4 | 4.875 |
| *Ruta graveolens* | 4 | 15.625 |
| *Santolina chamaecyparissus* | 4 | 5.625 |
| *Sinapis arvensis* | 4 | 9 |
| *Verbena rigida* | 4 | 6.875 |
| *Weigela florida* | 4 | 6.875 |
| *Zinnea haageana* | 4 | 3.625 |
| *Alcea rosea* | 3 | 6.666667 |
| *Anemone hipehensis* | 3 | 9.166667 |
| *Brassica spp.* | 3 | 4.166667 |
| *Centaurea montana* | 3 | 4.166667 |
| *Dianthus sp.* | 3 | 2.5 |
| *Eruca vesicaria* | 3 | 18.66667 |
| *Gaillardia pulchella* | 3 | 2.5 |
| *Helianthus tuberosus* | 3 | 2.333333 |
| *Hypericum perforatum* | 3 | 7.166667 |
| *Hypericum scouleri* | 3 | 5.666667 |
| *Lavandula angustifolia* | 3 | 5.5 |
| *Melilotus albus* | 3 | 6.5 |
| *Mentha spicata* | 3 | 10 |
| *Ocimum basilicum* | 3 | 26.66667 |
| *Origanum vulgare* | 3 | 3.333333 |
| *Pelargonium zonale* | 3 | 4.166667 |
| *Perovskia atriplicifolia* | 3 | 4.166667 |
| *Potentilla recta* | 3 | 13.33333 |
| *Rudbeckia fulgida* | 3 | 4.666667 |
| *Satureja hortensis* | 3 | 11.66667 |
| *Solanum dulcamara* | 3 | 3.333333 |
| *Zinnea elegans* | 3 | 4.75 |
| *Allium tuberosum* | 2 | 6 |
| *Amaranthus hybridus* | 2 | 10 |
| *Anemone sylvestris* | 2 | 15 |
| *Arabsis alpina* | 2 | 8.5 |
| *Brassicaceae unknown* | 2 | 9 |
| *Calendula arvensis* | 2 | 3 |
| *Calystegia sepium* | 2 | 2.5 |
| *Capsicum annuum* | 2 | 3 |
| *Chaenomeles japonica* | 2 | 4 |
| *Chelone obliqua* | 2 | 3.25 |
| *Chrysanthemum superbum* | 2 | 34 |
| *Circium vulgare* | 2 | 21 |
| *Dianthus armeria* | 2 | 3.75 |
| *Dianthus plumarius* | 2 | 2.5 |
| *Gladiolus communis* | 2 | 2 |
| *Glebionis segetum* | 2 | 5 |
| *Gypsophila elegans* | 2 | 4.75 |
| *Heliotropium arborescens* | 2 | 12.5 |
| *Hemerocallis fulva* | 2 | 2.25 |
| *Hydrangea arborescens* | 2 | 12.5 |
| *Hylotelephium telephium* | 2 | 10 |
| *Impatiens hawkeri* | 2 | 3.75 |
| *Ipomoea purpurea* | 2 | 6 |
| *Leucanthemum vulgare* | 2 | 17 |
| *Matricaria chamomilla* | 2 | 2.5 |
| *Nasturtium officinale* | 2 | 2 |
| *Oenothera biennis* | 2 | 2.5 |
| *Phaseolus sp.* | 2 | 12.5 |
| *Rubus idaeus* | 2 | 3 |
| *Salvia rosmarinus* | 2 | 3 |
| *Satureja montana* | 2 | 11 |
| *Sedum acre* | 2 | 3.75 |
| *Sonchus oleraceus* | 2 | 5 |
| *Symphytum officinale* | 2 | 3.75 |
| *Vicia faba* | 2 | 48 |
| *Alliaria petiolata* | 1 | 2.5 |
| *Allium ampeloprasum* | 1 | 2.5 |
| *Allium schoenoprasum* | 1 | 2.5 |
| *Anthriscus cerefolium* | 1 | 12 |
| *Arctium lappa* | 1 | 2.5 |
| *Astrantia major* | 1 | 2.5 |
| *Begonia cucullata* | 1 | 5 |
| *Borage officinalis* | 1 | 5 |
| *Calendula officinalis* | 1 | 2.5 |
| *Campanula persicifolia* | 1 | 2.5 |
| *Campsis radicans* | 1 | 5 |
| *Canna indica* | 1 | 5 |
| *Centaurea cyanus* | 1 | 5 |
| *Cerastium arvense* | 1 | 5 |
| *Chamaesyce hypericifolia* | 1 | 2 |
| *Chelidonium majus* | 1 | 10 |
| *Chicorium intybus* | 1 | 2.5 |
| *Conoclinium coelestimum* | 1 | 2.5 |
| *Convolvulus arvensis* | 1 | 9 |
| *Coreopsis tinctoria* | 1 | 5 |
| *Corepsis tinctoria* | 1 | 5 |
| *Coriandrum sativum* | 1 | 7 |
| *Crepis rubra* | 1 | 2.5 |
| *Crocosmia aurea* | 1 | 2.5 |
| *Cucumis sativus* | 1 | 7 |
| *Digitalis purpurea* | 1 | 4 |
| *Digitalis purpurea albiflora* | 1 | 2.5 |
| *Echinacea purpurea* | 1 | 4 |
| *Erysimum cheiri* | 1 | 7 |
| *Erysimum odoratum* | 1 | 5 |
| *Erysiumum cheiri* | 1 | 2.5 |
| *Eschscholtzia californica* | 1 | 5 |
| *Filipendula rubra* | 1 | 2 |
| *Fragaria x ananassa* | 1 | 2.5 |
| *Galinsoga parviflora* | 1 | 10 |
| *Galium asprellum* | 1 | 2.5 |
| *Galium mollugo* | 1 | 4 |
| *Geranium sanguineum* | 1 | 5 |
| *Geranium sylvaticum* | 1 | 2.5 |
| *Gerbera sp.* | 1 | 5 |
| *Gomphrena globosa* | 1 | 2.5 |
| *Helenium autumnale* | 1 | 5 |
| *Heliopsis helianthoides* | 1 | 7 |
| *Hosta plantaginea* | 1 | 1 |
| *Hypochaeris radicata* | 1 | 30 |
| *Impatiens balsamina* | 1 | 20 |
| *Lamium maculatum* | 1 | 2 |
| *Leonorus cardiaca* | 1 | 2 |
| *Liatris aspera* | 1 | 10 |
| *Liatris spicata* | 1 | 15 |
| *Lupinus sp.* | 1 | 2.5 |
| *Lycium barbarum* | 1 | 2 |
| *Lythrum salicaria* | 1 | 2.5 |
| *Malcomia spp.* | 1 | 4 |
| *Malva alcea* | 1 | 2 |
| *Malva officinalis* | 1 | 2.5 |
| *Malva sylvestris* | 1 | 12 |
| *Melilotus officinalis* | 1 | 5 |
| *Monarda didyma* | 1 | 8 |
| *Myosotis sp.* | 1 | 5 |
| *Nepeta racemosa* | 1 | 7 |
| *Oenothera fructicosa* | 1 | 2.5 |
| *Oenothera fruticosa* | 1 | 20 |
| *Origanum satirum* | 1 | 20 |
| *Oxalis stricta* | 1 | 4 |
| *Papaver rhoeas* | 1 | 4 |
| *Papaver somniferum* | 1 | 5 |
| *Persicaria maculosa* | 1 | 5 |
| *Petroselinum crispum* | 1 | 5 |
| *Petunia axillaris* | 1 | 4 |
| *Petunia violacea* | 1 | 5 |
| *Petunia x hybrida* | 1 | 2.5 |
| *Phaseolus coccineus* | 1 | 2.5 |
| *Phaseolus vulgaris* | 1 | 4 |
| *Physostegia virginiana* | 1 | 4 |
| *Rorippa sylvestris* | 1 | 5 |
| *Rosa gallica* | 1 | 5 |
| *Rosa rugosa* | 1 | 10 |
| *Rosa sp.* | 1 | 5 |
| *Rubus allegheniensis* | 1 | 8 |
| *Rubus idaeus var. strigosus* | 1 | 10 |
| *Salvia farinacea* | 1 | 2 |
| *Saponaria officinalis* | 1 | 70 |
| *Sedum sp.* | 1 | 10 |
| *Sempervivum tectorum* | 1 | 4 |
| *Senecio vulgaris* | 1 | 2.5 |
| *Silene vulgaris* | 1 | 4 |
| *Solidago canadensis* | 1 | 10 |
| *Sonchus asper* | 1 | 2.5 |
| *Spiraea japonica* | 1 | 5 |
| *Spirea x vanhouttei* | 1 | 10 |
| *Tagetes erecta* | 1 | 5 |
| *Tragopogon pratensis* | 1 | 24 |
| *Trifolium dubium* | 1 | 4 |
| *Vicia cracca* | 1 | 15 |
| *Vicia villosa* | 1 | 4 |
| **Total** | 246 spp. 1370 indiv | |
